# Avenues to molecular imaging of dying cells: Focus on cancer

**DOI:** 10.1002/med.21495

**Published:** 2018-03-12

**Authors:** Anna A. Rybczynska, Hendrikus H. Boersma, Steven de Jong, Jourik A. Gietema, Walter Noordzij, Rudi A. J. O. Dierckx, Philip H. Elsinga, Aren van Waarde

**Affiliations:** ^1^ Molecular Imaging Center, Department of Nuclear Medicine and Molecular Imaging University of Groningen, University Medical Center Groningen Groningen the Netherlands; ^2^ Department of Genetics University of Groningen Groningen the Netherlands; ^3^ Department of Clinical Pharmacy & Pharmacology University of Groningen Groningen the Netherlands; ^4^ Department of Medical Oncology University of Groningen Groningen the Netherlands; ^5^ Department of Nuclear Medicine Ghent University Ghent Belgium

**Keywords:** apoptosis, early treatment response, necrosis, positron emission tomography (PET), single photon emission computed tomography (SPECT)

## Abstract

Successful treatment of cancer patients requires balancing of the dose, timing, and type of therapeutic regimen. Detection of increased cell death may serve as a predictor of the eventual therapeutic success. Imaging of cell death may thus lead to early identification of treatment responders and nonresponders, and to “patient‐tailored therapy.” Cell death in organs and tissues of the human body can be visualized, using positron emission tomography or single‐photon emission computed tomography, although unsolved problems remain concerning target selection, tracer pharmacokinetics, target‐to‐nontarget ratio, and spatial and temporal resolution of the scans. Phosphatidylserine exposure by dying cells has been the most extensively studied imaging target. However, visualization of this process with radiolabeled Annexin A5 has not become routine in the clinical setting. Classification of death modes is no longer based only on cell morphology but also on biochemistry, and apoptosis is no longer found to be the preponderant mechanism of cell death after antitumor therapy, as was earlier believed. These conceptual changes have affected radiochemical efforts. Novel probes targeting changes in membrane permeability, cytoplasmic pH, mitochondrial membrane potential, or caspase activation have recently been explored. In this review, we discuss molecular changes in tumors which can be targeted to visualize cell death and we propose promising biomarkers for future exploration.

**Abbreviations**
γH2AX: phosphorylated X isoform of the histone H2A
ABC: ATP‐binding cassette
ApoPep‐1: apoptosis‐targeting peptide‐1
ATP: adenosine 5′‐triphosphate
ATR kinase: ataxia telangiectasia and Rad3‐related threonine serine kinase
Bcl‐2: B‐cell lymphoma 2
Caspase: cysteine‐aspartic protease
CytC: Cytochrome C
DDC: *N*,*N*’‐didansyl‐L‐cystine
DPA: dipicolylamine
ER: endoplasmic reticulum
FBnTP: fluorobenzyl triphenyl phosphonium
LysoPS: lyso‐phosphatidylserine
mAb: monoclonal antibody
MIAPaCa‐2: human pancreatic carcinoma cell line
mibi: methoxyisobutylisonitrile
ML: malonic acid
MMP: mitochondrial membrane potential
MW: molecular weight
PARP‐1: poly (ADP‐ribose) polymerase 1
PE: phosphatidylethanolamine
PET: positron emission tomography
PKC: protein kinase C
PS: phosphatidylserine
SA: streptavidin
SPECT: single photon emission computed tomography
TNF: tumor necrosis factor
TPP: tetraphenylphosphonium
TRAIL: TNF‐related apoptosis inducing ligand
z‐YVAD‐fmk: caspase‐1 inhibitor

## 1 INTRODUCTION

A living organism can be considered as a complicated machine, which requires constant maintenance, modernization, and restructuring or reconstruction. Subunits of the organism, such as cells, are continuously produced, exploited, altered, utilized and exchanged. Billions of cells die daily as a part of natural processes in the adult human body, and even more cells die during embryonic development. Under physiological conditions, superfluous, dangerous, or damaged cells are killed and dismantled in a discrete and highly orchestrated manner. For instance, squamous epithelial cells are removed via cornification,^1^ Müllerian duct in males or Wolffian duct in females via apoptosis, and pronephric kidney tubes also via apoptosis.^2^, ^3^ A mainstay of the body's homeostasis is a proper decision on cellular fate: death or survival.

It is thus not surprising that perturbations of cell death processes are an underlying factor of many pathologic conditions. Cell death is enhanced in ischemia,^4^ sepsis,^5^ type‐1 diabetes,^6^ transplant rejection,^7^ neurodegenerative disorders,^8^ and autoimmunity (e.g., AIDS).^9^ In contrast, reduced cell death is observed in persistent inflammation (as occurs in chronic obstructive pulmonary disease and asthma),^10^, ^11^ autoimmunity (e.g., rheumatoid arthritis),^12^ and cancer.^13^ With nondestructive and minimally invasive medical imaging techniques like PET (positron emission tomography) and SPECT (single photon emission computed tomography), cell death in organs and tissues of the human body can be visualized and quantified. Such quantification may be important in cancer treatment, since monitoring of the increase in cell death early after the onset of antitumor therapy can serve as a predictor of the eventual therapeutic outcome.

In the following review, we describe molecular changes in tumors related to cell death and we provide an overview of the wide range of PET and SPECT tracers which have been developed to monitor these changes. We discuss the potential and the limitations of the existing tracers and we propose some promising biomarkers of dying cells which deserve to be explored in future imaging research.

### 1.1 Canonical classification of cell death modes

There are many ways for a cell to die. In recent years our concepts of cell death have changed. In this chapter, we first describe the canonical classification of cell death modes and we subsequently summarize new observations which have led to a revised classification.

The classical concept of cell death (proposed in 1973) is based on morphologic features of dying cells and makes a distinction between three death types: apoptosis (type I), autophagic cell death (type II), and necrosis (type III) (see Table 1).^14^ Even nowadays, cell death is still frequently classified in these three subroutines. Apoptosis and autophagy are considered as “regulated” and necrosis as “accidental” cell death.^15^

**Table 1:** Morphological classification of cell death

Apoptosis (Type I)	Autophagic cell death (Type II)	Necrosis (Type III)
Affects an individual cell	Affects an individual cell	Affects a group of cells
Cell rounding, shrinkage and detachment	Cytoplasmic vacuolization	Increased cell volume (oncosis), translucent and vacuolized cytoplasm
Cell membrane blebbing and shedding of apoptotic bodies, but membrane intact	Cell membrane intact	Cell membrane breakdown
Maintained organelles and cytoplasm condensation	Degradation of Golgi, polyribosomes and ER	Swollen organelles and cytoplasm
Chromatin condensation (pyknosis)	No/partial chromatin condensation	Chromatin condensation into small, irregular patches (karyolysis)
Nuclear fragmentation (karyorrhexis)	Appearance of autophagosomes and autolysosomes	Dilatation of the nuclear membrane
DNA fragmentation	Late DNA fragmentation	Late DNA fragmentation (after cell lysis)
Presence of phagocytosis, generally anti‐inflammatory	No/little phagocytosis	Generally absence of phagocytosis, often pro‐inflammatory

#### 1.1.1 Apoptosis

Apoptosis was considered to be a noninflammatory, highly orchestrated, and inherently controlled process. Since its identification in 1972,^16^ apoptosis has been the most investigated type of cell death. Apoptosis can be activated by intra‐ or extracellular stimuli and is then coined as “intrinsic” or “extrinsic” apoptosis. Both these apoptotic scenarios include extensive cellular remodeling by activated cysteine–aspartic proteases, called “caspases” (for more information, see 2.4.).

In intrinsic apoptosis, stimuli such as DNA damage and hypoxia lead to swelling or permeabilization of the mitochondrial outer membrane, dissipation of the mitochondrial membrane potential (MMP), and release of various apoptotic effectors. Apoptotic effectors serve either as activators of the proapoptotic cascade or inhibitors of the pro‐survival cascade. Apoptosome complex forming compounds, such as caspase‐9, cytochrome c (CytC), apoptotic peptidase‐activating factor 1, deoxy‐adenosine 5′‐triphosphate (deoxy‐ATP), and second mitochondria‐derived activator of caspases (second mitochondria‐derived activator of caspase/direct inhibitor of apoptosis‐binding protein with low Isoelectric point (pI)) belong to the activator category, whereas B‐cell lymphoma 2 (Bcl‐2) family members and inhibitors of apoptosis proteins are in the inhibitor class.^17^, ^18^, ^19^

Extrinsic apoptosis is activated by the appearance of multiple members of a tumor necrosis factor (TNF) family of ligands via death receptors, or by the disappearance of specific ligands for dependence receptors. Death receptor ligands include TNFα, first apoptosis signal ligand which binds to the Fas receptor, and TNF‐related apoptosis inducing ligand (TRAIL), which interacts with the TRAIL receptors.^20^, ^21^ An example of a ligand for a dependence receptor is netrin‐1, which binds to the uncoordinated movement receptor gene 5B (mutations in this gene result in uncoordinated movement of Caenorhabditis elegans) receptor.^22^ Main effectors activating the proapoptotic cascade are death‐inducing signaling complex‐forming: Fas‐associated protein with death domain, caspase‐8 and caspase‐10, whereas main effectors inhibiting the proapoptotic cascade are cellular Fas‐associated protein with death domain‐like IL‐1β‐converting enzyme‐inhibitory protein and x‐linked inhibitor of apoptosis protein.^23^, ^24^, ^25^ Extrinsic apoptosis is frequently linked to the response of the immune system to abnormalities.

Under certain circumstances (e.g., high x‐linked inhibitor of apoptosis protein expression levels), components of the intrinsic apoptosis machinery can also become activated during extrinsic apoptosis. This interrelation of extrinsic and intrinsic signaling is mediated by a proapoptotic Bcl‐2 member, Bcl‐2 homology domain 3 interacting‐domain death agonist, and serves for amplification of an apoptotic signal downstream death receptors.^26^ Furthermore, intrinsic and extrinsic apoptosis converge through caspase‐9 and caspase‐8, which leads to activation of caspase‐3 and cellular disassembly from within. Activation of caspase‐3 is followed by cleavage of cytosolic and nuclear proteins, DNA fragmentation, cross‐linking of proteins, formation of apoptotic bodies, expression of ligands for phagocytic cell receptors, and removal of apoptotic cells by phagocytosis.^27^

Evasion of cell death is considered to play an important role in oncogenesis and in development of treatment resistance in cancer.^28^ One example of apoptosis evasion is a decrease in p53 signaling. P53 is a tumor suppressor protein, which can regulate the cell cycle and can induce cancer cell apoptosis in response to diverse stressful stimuli. Frequent mutations in the *TP53* gene and/or defects in the p53 signaling pathway (e.g., upregulation of the p53 inhibitor mouse double minute 2, mouse double minute 2 homolog [E3 ubiquitin‐protein ligase]) result in uncontrolled proliferation and a brake on apoptosis. This may have a subsequent impact on both initiation of oncogenesis and development of treatment resistance. Although apoptosis is the best‐characterized cell death mechanism, in many cancers it is not the main cause of cell loss induced by DNA damaging agents.^28^

#### 1.1.2 Autophagic cell death

Autophagy is a natural, regulated process for disassembly of dysfunctional or damaged cellular organelles and proteins. Such damaged components are contained inside a double‐membrane vesicle called an autophagosome. After fusion of an autophagosome and a lysosome to an autolysosome, the contents of the organelle are digested by acidic lysosomal hydrolases.^29^

Even today, there is much controversy on the question whether in vivo autophagy is a type of cell death or fulfills a pro‐survival function, for example, by limiting cell constituents during nutrient starvation. This question is raised because most inhibitors of autophagy *accelerate* (and not retard) cell death.^30^, ^31^, ^32^, ^33^, ^34^ For this reason, autophagic cell death has now been defined as cell death inhibited by inactivation of autophagy genes or by autophagy inhibitors, such as 3MA, rather than cell death judged by simple morphological classification.^35^ This definition is based on studies which have elucidated molecular mechanisms of autophagic cell death.^36^, ^37^ Tissue‐specific knockout models of genes controlling autophagy in mice have provided much information about the role of autophagy in the development and differentiation of mammalian tissues and organs.^38^ In some tissues (e.g., mouse liver) autophagy seems to suppress tumorigenesis,^39^ but in most cases, autophagy facilitates the formation of tumors and increases tumor growth and aggressiveness.^40^ Autophagy seems to be particularly induced when cancers progress to metastasis.^41^ Inhibitors of autophagy may thus be useful as adjuvants in cancer therapy.

#### 1.1.3 Necrosis

Necrosis is the consequence of irreversible damage to cells caused by factors such as mechanical trauma, infections, toxins, and shortage of oxygen and nutrients. Necrosis is traditionally thought to be an uncontrollable and accidental type of cell death, which is highly immunogenic and elicits an inflammatory response due to leakage of cytosolic contents. It was considered the death mode of cells which displayed no characteristics of apoptosis. In most cases necrosis affects not a single cell but spreads over a group of cells, as in gangrene or ischemia. Morphologic features of necrosis are listed in Table 1. At the biochemical level, necrosis is accompanied by a massive production of reactive oxygen species and reactive nitrogen species, besides a marked drop of cellular ATP.^35^

About 10 years ago, studies on genes that could control necrosis led to the conclusion that a regulated form of necrosis must exist. Regulated necrosis (“necroptosis”) can occur as the result of activation of death receptors, for example, by TNF, first apoptosis signal ligand, or TRAIL,^42^ and is controlled by two key regulators:TNF receptor‐associated factor 2 and receptor‐interacting protein kinases 1 and 3.^35^, ^43^ Besides the activation of death receptors, necroptosis requires inhibition of the apoptotic signaling.^44^ This type of necrosis occurs not only in disease (e.g., in systemic inflammatory response syndrome), but also in normal physiology (e.g., in immunologically silent maintenance of T‐cell homeostasis).^45^, ^46^ In cancer, necrosis occurs when rapid tumor growth is accompanied by insufficient vascularization or the cancer cell population becomes very dense.^47^ It can also be a consequence of successful immunotherapy, for example, with oncolytic viruses.^48^ The triggering of nonapoptotic cell death modes, such as regulated necrosis, is currently explored for treatment of apoptosis‐resistant cancer cells.^49^ However, clinical application of regulated necrosis in cancer treatment has not yet been achieved.

### 1.2 Revised classification of cell death modes

Canonical (morphologic) features of a particular cell death mode can be inhibited while death is only deferred.^15^ Under certain circumstances, a dying cell can even switch between different cell death programs, for example, the response to DNA damage changes from apoptosis to mitotic catastrophe in p53‐expressing ovarian cancer treated with cisplatin versus cisplatin and checkpoint kinase 2 (required for checkpoint‐mediated cell cycle arrest) inhibitor^50^, ^51^, ^52^ or from apoptosis to (secondary) necrosis in conditions of insufficient phagocytosis. This suggests that an interplay and/or a fluidic switch may exist between various types of cell death.^53^ Apparently, cell death may differ not only in its main morphologic features but also in biochemical features, cell types involved, and activating mechanisms. Moreover, morphologic features are hardly quantifiable and do not take functional, biochemical, and immunological variables into account. Therefore, scientists have shifted from a morphological to a biochemical classification of cell death.^35^ As a consequence, the canonical distinction of three different cell death modes has been revised and expanded to comprise 14 subroutines (see Table 2), of which ten play a proven role in treatment‐induced cancer cell death.^15^, ^35^, ^54^ These include: apoptosis (divided into: intrinsic caspase‐dependent, intrinsic caspase‐independent, extrinsic by death receptors, extrinsic by dependence receptors), unregulated necrosis, regulated necrosis (necroptosis), pyroptosis, autophagic cell death, mitotic catastrophe, and anoikis. It is still hotly debated whether some of these processes (e.g., autophagic cell death and mitotic catastrophe) are true subroutines or associated phenomena preceding cell death (for more information, see).^35^, ^55^ Furthermore, it is still not clear which of these subroutines predominates in cell death induced by antitumor treatment and which route should be activated for the most effective treatment of a particular type of cancer.^28^ Nevertheless, this new classification of cell death allows a better separation of molecular pathways and the linking of pathways to functional consequences.

**Table 2:** Revised classification of cell death modes and their characteristics

Cell death mode	PS exposure	Decrease in MMP	Cell membrane rupture	Active caspases	DNA fragmentation/hydrolysis	Hallmarks/markers	Inducer (example)	Inhibitor (example)
Caspase‐dependent intrinsic apoptosis* ^,^ 35, 282	++	++	+	++	++	Bak (BCl‐2 homologous antagonist/killer), Bax (BCl‐2‐associated X protein) activation CytC (second mitochondria‐derived activator of caspase). SMAC/DIABLO (direct inhibitor of apoptosis‐binding protein with low pI), HTRA2 release from mitochondria Caspase‐9, ‐3, ‐6, ‐7 activation	Raptinal Cadmium	z‐LEHD‐fmk (caspase‐8 inhibitor) Cyclosporin A
Caspase‐independent intrinsic apoptosis* ^,^ 35, 283	++	++	+	−	++	BNIP‐3 (BCL2/adenovirus E1B 19 kDa protein‐interacting protein 3) overexpression EndoG (endonuclease G), AIF, HTRA2 release from mitochondria ROS production	Cadmium	Cyclosporin A
Extrinsic apoptosis by death receptors* ^,^ 35	++	+	+	++	++	Death receptor activation Caspase‐8, ‐10, ‐3, ‐7 activation CytC release, BID (Bcl‐2 homology domain 3 interacting‐domain death agonist) cleavage	FasL (first apoptosis signal ligand)	z‐IETD‐fmk (caspase‐8 inhibitor)
Extrinsic apoptosis by dependence receptors* ^,^ 35, 284	++	+	+	++	++	Dependence receptor activation (patched, uncoordinated movement receptor gene 5A, DCC [deleted in Colorectal Cancer gene]) Caspase‐9, ‐3, ‐7 activation	Suboptimal netrin concentration	z‐LEHD‐fmk (caspase‐8 inhibitor)
Autophagic cell death* ^,^ 29, 35	++	+	−	−	−/+	PE conjugated LC3 (LC3‐II) Beclin‐1 accumulation SQSTM1 (sequestosome1 [ubiquitin‐binding protein p62])/p62 degradation	Rapamycin	3‐methyl‐adenine Atg5, Atg7, Beclin‐1 VPS34 (Class III phosphoinositide 3‐kinase) genetic inactivation
Necroptosis* ^,^ 285	−	++	++	−	+	RIP1, RIP3 activation MLKL (mixed lineage kinase domain like pseudokinase) activation	TNF	Caspase‐8 Necrostatin‐1
Pyroptosis* ^,^ 286, 287	++	++	++	++	++	Caspase‐1, ‐4, ‐5 activation IL1ß (interleukin 1 beta), IL18 (interleukin 18) secretion Gasdermin D cleavage	LPS (lipopolysaccharide)	z‐YVAD‐fmk (caspase‐1 inhibitor)
Mitotic catastrophe* ^,^ 288, 289	++	+	++	−	+	Cyclin B accumulation CDK1 (cyclin‐dependent kinase 1) activation	Cytochalasin D Trichostatin A	Survivin
Anoikis* ^,^ 290, 291	++	++	+	++	++	Epidermal growth factor receptor downregulation BIM overexpression BMF (Bcl2‐modifying factor) phosphorylation JNK (c‐Jun N‐terminal kinases) activation	Cell disengagement from the extracellular matrix	bFGF (basic fibroblast growth factor) z‐VAD‐fmk (cell‐permeable, irreversible pan‐caspase inhibitor)
Cornification^292^	n.d.	+	−	+	++	Transglutaminases, caspase‐4 activation		
Netosis^293^	+	++	++	−	−	NADPH (β‐nicotinamide adenine dinucleotide, reduced) oxidase activation ROS production	PMA (phorbol 12‐myristate 13‐acetate)	Diphenyl iodide
Parthanatos^294^	+	++	++	−	++	PARP1 activity increased PAR accumulation Mitochrondria release AIF AIF translocates to nucleus MIF activity increased MIF translocates to nucleus	MNNG (methylnitronitrosoguanidine)	Niraparib
Entosis^295^	−	−	−	−	(+)	RhoA (Ras homolog gene family, member A), ROCK1/2 (Rho‐associated, coiled‐coil containing protein kinase 1/2) activation AMPK (5′‐AMP‐activated protein kinase) increased E‐cadherin increased LC3 lipidation	Glucose starvation AICAR (5‐aminoimidazole‐4‐carboxamide ribonucleotide)	Y‐27632 (selective inhibitor of Rho‐associated protein kinase p160ROCK) Compound C
Necrosis‐oncosis* ^,^ 296, 297	−	−	++	−	−/+	Rapid decline of intracellular ATP Reduced activity of ion pumps (Ca^2+^, Na^+^/K^+^ ATPases)	H_2_O_2_	
Ferroptosis^298^	−	++	−	−	−	Reduced cysteine uptake Production of ROS GPX4 (glutathione peroxidase 4) inhibition Glutathione depletion	Erastin	Ferrostatin‐1

An asterisk (*) indicates cell death modes known to apply to therapy‐induced cancer cell death, ++ = process strongly increased, + = process increased, − = process not increased, n.d. = activity of process not determined.

AIF, apoptosis‐inducing factor; Atg, genes controlling autophagy; fmk, fluoromethyl ketone; HTRA2, HTrA serine peptidase 2; LC3, microtubule‐associated protein 1A/1B‐light chain 3; MIF, macrophage migration inhibiting factor; ROS, reactive oxygen species.

In order to properly classify cell death, several parameters should be determined since many biochemical processes that were initially considered to be hallmarks of apoptosis appear also in other death modes (Table 2). Despite this complexity, five main biochemical parameters appear to define dying cells: (1) changes of membrane asymmetry (exposure of phosphatidylethanolamine [PE] and phosphatidylserine [PS]), (2) loss of transmembrane potential, (3) permeabilization of the mitochondrial membrane with associated potential changes, (4) increased proteolysis, and (5) DNA fragmentation. We will discuss these in the following chapter.

## 2 HALLMARKS OF CELL DEATH

**Figure 1:**
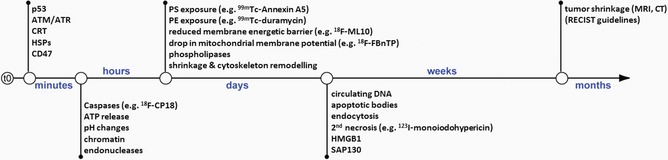
Physiologic, molecular, and morphologic events during the time‐course of cell death

As listed in Table 2, each of the five characteristics of apoptosis occurs in more than one cell death mode. However, the order of their appearance on the scenario of cell death is generally well preserved (see Figure 1).

### 2.1 Changes in membrane asymmetry

The cell membrane is a highly specialized bilayer of asymmetrically distributed phospholipids. In the resting state, cationic phospholipids prevail in the outer, and anionic phospholipids in the inner membrane leaflet. The cell membrane functions as: a *barrier* (allowing passage of only a selected set of molecules), an *organizer* (assembling, co‐localizing, and controlling activity of signaling components), and a *sensor and communicator* (processing and conducting signals between the cell and its environment).^56^ Multiple cellular activities are accompanied by changes in morphology or composition of the cell membrane. These activities include the regulation of immunity, coagulation and bone formation, for example, by changing the conformation, interactions, localization, and destination of proteins.^57^, ^58^, ^59^, ^60^, ^61^

A hallmark of apoptosis is the disturbance of membrane asymmetry, and specifically, the translocation of phospholipids, such as PE and PS, from the inner to the outer leaflet of the membrane. Under basal conditions, PE is predominantly and PS is almost exclusively confined to the inner leaflet of the cell membrane (in erythrocytes, 80–85% and >96%, respectively).^62^ Once on the cell surface, exposed PE may regulate actin‐dependent blebbing and the formation of apoptotic bodies,^63^, ^64^, ^65^ whereas exposed PS serves as a recognition and docking site, for example, for phagocytes, and facilitates the removal of apoptotic cells.^66^, ^67^, ^68^

Although disturbance of membrane asymmetry is a feature of apoptosis, disturbed asymmetry also appears early after activation of other cell death modes, such as anoikis, autophagic cell death, pyroptosis and mitotic catastrophe (Table 2).^69^, ^70^, ^71^, ^72^ In death modes such as necrosis, PE and PS may become accessible only at later time points, when cell membrane integrity has been lost.^73^

#### 2.1.1 Phosphatidylethanolamine exposure

PE is a neutral (zwitterionic) molecule which accounts for 40–50% of total membrane phospholipids.^74^ Most PE molecules are cone‐shaped and do not organize themselves into membrane bilayers in an artificial setting, but rather form monolayers,^75^ although PE is kept in bilayer configuration in biological membranes by interaction with other phospholipids. This feature enables PE to “coat” lipophilic regions of membrane proteins and to participate in membrane fusion and fission. In hepatocytes, the presence of PE in the bilayer was shown to result in a less tight packing of the membrane lipids and increased membrane permeability.^76^

The dynamics of PE play a role in membrane reorganization during cytokinesis,^77^, ^78^ stress and apoptosis,^63^, ^79^ and possibly also in hemostasis^80^ and the physiology of the mitochondrial inner membrane.^81^, ^82^ The appearance of PE on the surface may be a more sensitive biomarker of cell stress than PS, since PE is more abundant than PS and could deliver a stronger signal.^64^, ^82^ Moreover, PE is present on the luminal surface of tumor blood vessels. Exposed PE in the vessel wall may represent a biomarker for imaging response to antivascular cancer therapy.^64^

#### 2.1.2 Phosphatidylserine exposure

PS is an anionic molecule accounting for 2–10% of the total membrane phospholipids.^83^, ^84^ It has a cylindrical shape, which promotes formation of membrane bilayers. However, at elevated pH or [Ca^2+^], PS can adopt a conical shape to form hexagonal membrane structures.^85^, ^86^, ^87^ PS is inhomogenously distributed in the plasma membrane, forming 11 nm clusters.^88^

As mentioned above, PS exposure is a hallmark of apoptosis and an “eat me” signal for phagocytosis of dying cells. Many biochemical assays (e.g., in vitro staining of cells with Annexin A5) use PS exposure as a marker of apoptosis. Since annexin is not able to selectively identify apoptosis, Annexin A5 is then used in combination with propidium iodide to identify necrotic cells from apoptotic cells. Early in apoptosis, 10^6^–10^9^ PS molecules become accessible to Annexin A5 after translocation to the outer leaflet of the cell membrane.^89^, ^90^

However, PS exposure also occurs in normal physiology. For example, binding of proteins to intracellular PS can localize their signaling pathways to the proximity of the cell membrane (e.g., PS–PKC [protein kinase C] interaction)^91^, ^92^ and/or can promote membrane fusion and fission (e.g., PS‐synaptotagmin‐I interaction).^93^ PS exposure plays a role in physiological processes such as cell activation (platelets in clotting cascade, lymphocytes in immune response), membrane fusion in phagocytosis,^94^ release of membrane‐encapsulated nuclei during maturation of erythroblasts,^95^ and cellular stress responses.^96^, ^97^ Up to 50% of blood vessels in untreated tumors are positive for exposed PS, likely due to oxidative stress in their environment.^98^, ^99^ This fraction generally increases after anticancer treatment.^100^

In recent years it has become apparent that different forms of PS play unique and important signaling roles in the cell. Oxidized PS was shown to promote recognition of apoptotic cells by macrophages *via* interaction with CD36 (cluster of differentiation 36 [fatty acid translocase])^101^ or the bridging protein lactadherin (aka milk fat globule‐epidermal growth factor 8 protein, MFGE8).^102^ Up to 20% of the PS in neutrophils is endogenously converted to PS with only a single acyl chain lyso‐phosphatidylserine (lysoPS), in a β‐nicotinamide adenine dinucleotide (reduced) oxidase‐dependent manner. LysoPS plays a role in the clearance of PS‐expressing, nonapoptotic neutrophilic cells.^103^

1‐Lyso‐2‐acyl‐PS and 1‐acyl‐2‐lyso‐PS (PS with deletions of the first or second acyl chain) perform different cellular functions.^104^, ^105^, ^106^ 1‐Lyso‐2‐acyl‐PS can signal platelet degranulation, mast cell activation, and T‐cell growth suppression; and 1‐acyl‐2‐lyso‐PS may accompany histamine release from peritoneal mast cells and neuronal differentiation. However, our understanding of the role of different forms of lysoPS in cancer cell death is still rudimentary.

#### 2.1.3 Mechanism of PE and PS exposure

Currently, there are two models describing PS exposure during apoptosis: a recently proposed model of increased phospholipid vesicle trafficking (involving lysosomes,^107^ or bidirectional endosomes^108^) and a widely accepted model of disturbed phospholipid transport.^109^, ^110^, ^111^, ^112^

According to the first model, PS externalization reflects phospholipid vesicle trafficking between plasma membrane and cytoplasm rather than an activity of phospholipid transporters.^108^ This model is supported by the finding that PS externalization during apoptosis is derived from a newly synthesized pool, and the rate of PS synthesis is then ∼twofold increased.^113^, ^114^ Furthermore, altered lipid packing in shrinking cells can prompt PS exposure.^115^

According to the second model, localization of PE and PS is regulated by a common set of transporters, such as scramblases,^116^, ^117^ ATP‐binding cassette (ABC) transporters,^118^ and aminophospholipid translocases.^119^ Scramblases carry out Ca^2+^‐dependent bidirectional and nonspecific transport of phospholipids, whereas ATP‐dependent ABC transporters (floppases) and aminophospholipid translocases (flippases) transport PS and PE appropriately between the two leaflets of the cell membrane, that is, in outward or inward direction. The more specific localization of PS than PE to the intracellular leaflet under baseline conditions may be attributed to the fact that aminophospholipid translocases have a somewhat lower affinity for PE than for PS. It is generally accepted that apoptosis leads to deactivation of aminophospholipid translocases and activation of scramblases and ABC transporters.^109^, ^110^, ^111^, ^112^ Scramblases are activated by elevation of cytosolic Ca^2+^, an upstream event in, for example, apoptosis and blood coagulation. However, the identity of the transporters that are activated during cancer cell apoptosis has been the subject of a long debate.

The speed, strength, persistence, and reversibility of the signal are the best‐characterized features of PS exposure. Exposure of PS to the outer leaflet has been shown to occur within a few hours after induction of apoptosis.^120^ In human promyelocytic leukemia cells and Jurkat cells (immortalized line of human T lymphocytes) treated with various apoptosis inducers (e.g., anti‐Fas antibody or camptothecin), the content of PS in the outer leaflet increased 25–280‐fold (from <0.9 to >240 pmole/million cells).^67^, ^120^ At least an eightfold increase in externalized PS had to be reached to initiate phagocytosis of these cells, which is in line with the threshold model.^120^ In myocardial ischemia in mice, PS exposure on apoptotic cardiomyocytes was shown to persist for about 6 hours (hr) after reperfusion.^121^

The upstream signaling cascade leading to PE and PS externalization in apoptosis has also been examined. PS exposure is usually accompanied by other molecular events, such as caspase activation,^121^, ^122^, ^123^ cathepsin D activation,^124^ perturbed Ca^2+^ homeostasis,^125^, ^126^, ^127^, ^128^ and PKC activation.^129^, ^130^ Whether these processes may occur in parallel or are required in combination to initiate PE and PS exposure is not yet clear.^108^ A direct role of caspases in PS exposure during apoptosis has been suggested by the discovery of Kell blood group precursor‐related protein 8, which requires a caspase‐3 cleavage site to support presentation of PS on the surface of a dying cell followed by phagocytosis.^131^ In the human myeloid leukemia cell line KBM7, the P4‐ATPases ATPase phospholipid transporting, type 11C and cell division cycle protein 50A were shown to act as flippases and to transport aminophospholipids from the outer to the inner leaflet of the plasma membrane.^132^ ATPase phospholipid transporting, type 11C is a caspase substrate. Caspase‐mediated apoptotic exposure of PS is irreversible and leads to cellular engulfment by macrophages.

PS exposure is not under all circumstances closely related to cell death and phagocytic removal. PS can be exposed by viable cells, but is then likely an insufficient trigger for phagocytosis.^133^ However, blocking PS on dying cells can abrogate their clearance by phagocytosis. Therefore, phagocytes recognize cell surface PS on dying cells most likely only within strongly curved membrane areas (i.e., in blebs). However, little is known about membrane morphology surrounding exposed PE and PS and how these phospholipids are engaged by specific receptors, for example, lactadherin.^66^, ^134^, ^135^ Furthermore, several tumor cell lines have been identified that lack PS exposure during apoptosis^108^ and PS exposure can be reversible.^97^, ^121^, ^136^, ^137^

### 2.2 Loss of cellular transmembrane potential

Scrambling processes in early apoptosis reduce the pH of the external membrane leaflet and cytoplasm (acidification), and reduce the energy barrier of the cell membrane (depolarization).^138^, ^139^ The mechanism of cytoplasm acidification is not yet completely understood. A change in PS localization during apoptosis may affect the function of H^+^‐ATPases, increase proton (H^+^) transport across the cell membrane, and reduce cytoplasmic pH.^140^, ^141^ Under basal conditions the cytoplasm has a pH of about 7.2 which decreases by about 0.3 to 0.4 pH units in early apoptosis. This drop promotes the activity of important enzymes involved in cell death, such as proteases and DNase II.^142^ A loss of plasma membrane potential can be due to a change in cationic and anionic phospholipid distribution, an altered balance between extracellular Na^+^ and intracellular K^+^ (e.g., impaired function of Na^+^/K^+^‐ATPase) and export of intracellular Cl^−^. The impairment of Na^+^/K^+^ ATPase function in apoptotic cells was shown to be caspase‐dependent and coincided with mitochondrial depolarization.^143^

### 2.3 Change in mitochondrial transmembrane potential (Δψ_m_)

Ca^2+^ is a very powerful regulator of many biochemical processes. Therefore, its cellular concentration must be tightly controlled. Increases in cytoplasmic Ca^2+^ (e.g., caused by calcium release from the endoplasmic reticulum [ER]) can be resolved by mitochondria.^144^ Mitochondria are one of the largest stores of intracellular Ca^2+^ (after the ER), and centers of cellular energy production by oxidative phosphorylation. The functioning electron transport chain facilitates the creation of an electrochemical gradient (δpH) across the inner mitochondrial membrane and the creation of an MMP (Δψ_m_). The highly negative charge generated at the inner mitochondrial membrane by oxidative phosphorylation is strongly reduced when cells are energetically compromised and on their way to death. Certain apoptotic stimuli (e.g., ER stressors, death receptors, DNA damage) may cause a mitochondrial Ca^2+^ overload and spillage of Ca^2+^ into the cytoplasm. Ca^2+^ efflux is regulated by the Na^+^/Ca^2+^ exchanger and the permeability transition pore complex formed by proapoptotic Bcl‐2 family members. A disturbance in Ca^2+^ homeostasis and transition pore formation was shown to result in inhibition of oxidative phosphorylation and electron transport, dissipation of Δψ_m_ and/or generation of mitochondrial outer membrane permeability, a decrease in cellular ATP, release of proteins from the mitochondrial intermembrane space, and activation of cytoplasmic Ca^2+^‐dependent endonucleases.^145^, ^146^ Factors which are then released from mitochondria include ATP, reactive oxygen species, and facilitators of caspase‐9 activity, such as CytC, apoptosis‐inducing factor, and second mitochondria‐derived activator of caspase (see Section 1.1). The release of such factors is thought to be “a point‐of‐no‐return” in the apoptotic cascade.^147^, ^148^

Changes in mitochondrial transmembrane potential can be both the cause and a consequence of apoptosis. They are the cause if certain agents induce mitochondrial damage and downstream activation of caspase‐9, and a consequence if mitochondria amplify the apoptotic cascade downstream death receptors and caspase‐8 has already become activated. Depolarization (or, in rare cases, hyperpolarization) of the mitochondrial membrane occurs in response to a cellular insult.^149^, ^150^

Changes in Δψ_m_ are frequently monitored as an indicator of cell viability. Almost each form of cell death results in declined ψ_m,_ either at an earlier or a later stage, but an interesting study has shown that release of certain proapoptotic molecules (such as CytC) may occur in the absence of changes in mitochondrial outer membrane potential.^151^

### 2.4 Increased caspase proteolysis

Cell death is frequently mediated by a proteolytic cascade, in which caspases play a pivotal role. Caspases have been demonstrated to cleave as much as 5% of the cellular proteome during apoptosis.^152^, ^153^ The caspases are a family of enzymes with the ability to sever a myriad of peptides and proteins at residues C‐terminally to aspartate (Asp, D). They contain a catalytic Cys‐His pair with Cys285 acting as the nucleophile and His237 acting as the general base to abstract the proton from the catalytic Cys and promote the nucleophile. Caspases recognize and cleave proteins after the tetra‐peptide motif Asp‐x‐x‐Asp. The enzymes occur as dimers and are mostly present in the cytoplasmic compartment of the cell.

To date, at least 11 caspases (14 according to ref. 154) and 11 caspase‐encoding genes were identified in the human genome and proteome. Although these proteases are generally known as executioners of apoptosis, nonapoptotic activities have also been reported.^155^ Thus, they can be classified as apototic and nonapoptotic (inflammatory) caspases. The apoptotic caspases comprise apoptosis initiators (caspase‐2, ‐8, ‐9, and ‐10) and apoptosis executors (caspase‐3, ‐6, and ‐7). The executor caspases can cleave hundreds of substrates.^156^ Caspase‐3 is the main executer of apoptosis. Among its substrates are proteins participating in DNA repair (e.g., poly [ADP‐ribose] polymerase 1, PARP‐1), cytoskeletal proteins (e.g., fodrin), remodeling proteins (e.g., Rho‐associated, coiled‐coil containing protein kinase 1), and nuclear proteins (e.g., lamin B1). (Primarily) nonapoptotic caspases include caspase‐1, ‐4, ‐5, and ‐14.

In the absence of a demand for proteolytic activity, caspases are present in an inactive zymogen form (procaspases). Upon specific cellular insults, two procaspases are cleaved in a highly controlled manner into two small and two large subunits, assembled into a heterotetramer and activated. By cleaving a specific range of assigned protein substrates, caspases render a controlled loss, gain, functional change, or altered localization of client proteins. This in turn leads to the appearance of typical apoptotic characteristics, such as disturbance of cell membrane lipid asymmetry, cell shrinkage, nuclear chromatin condensation, and DNA fragmentation.

Synthetic caspase‐3/7 substrates should consist of at least five amino acid residues. Caspase substrates are selected based on protein primary, secondary, tertiary, and quaternary structure.^152^ The design of synthetic caspase substrates is based on the preference of caspases for individual peptide sequences (subsite preference).^157^

### 2.5 DNA fragmentation

DNA fragmentation is a major step of cellular disassembly. The process may be induced by cell death‐inducing factors (e.g., cytolytic T‐cells) or by irreparable errors or damage to DNA (e.g., radiation damage). Genomic DNA can be hydrolyzed either inside or outside a dying cell.^158^ DNA hydrolysis occurs at different time points and has a different pattern in different cell death modes.

Cleavage of DNA is executed by certain enzymes, DNA endonucleases, which are also known as DNases. These DNases are divided into three groups: (a) Ca^2+^/Mg^2+^ endonucleases (e.g., DNase I and DNAS1L3), (b) Mg^2+^ endonucleases (e.g., endonuclease G and DFF40/caspase‐activated DNase), and (c) cation‐independent/acid endonucleases (e.g., DNase II). The activity of these DNases is controlled by various means, such as protease activation (caspases or serine proteases), poly(ADP ribosylation), phosphorylation, or ubiquitination, and by physicochemical conditions, such as a change of cytoplasmic pH.^142^, ^159^ Activation of various DNases results in different DNA fragmentation patterns.

(Inter)nucleosomal DNA fragmentation yielding low molecular weight (MW) DNA fragments (“laddering pattern”) almost always accompanies apoptosis. Caspase‐activated DNase (present in extrinsic and intrinsic apoptosis) and endonuclease G (present in intrinsic apoptosis) produce various laddering patterns.^160^, ^161^, ^162^, ^163^, ^164^ The selection of a certain DNase seems to be stimulus‐ and cell type‐dependent. DNA is processed in two steps during apoptosis. In the early stage, DNA is cleaved into high MW fragments (50–300 kb). Here DNA condensation takes place. Subsequently, these molecules are further broken up into oligonucleosome‐sized fragments (repeats of 180–200 bp).^165^ Free DNA termini present as a consequence of apoptosis can be detected by a TdT‐mediated‐dUTP nick end labeling assay.^166^ However, DNA breaks detected by this assay need not to be a consequence of apoptosis. The TdT‐mediated‐dUTP nick end labeling assay cannot discriminate among apoptosis, necrosis, and autolytic cell death.

A more random form of DNA fragmentation, yielding a “smear pattern,” is observed in nonapoptotic cell death modes, such as necrosis, or cellular disassembly after phagocytosis. This pattern results from the activity of lysosomal DNases, for example, DNase II.

## 3 CELL DEATH IMAGING

Since the mechanisms underlying cell death are complex, the question arises how treatment‐induced cell death, for example, in cancer, should be quantified with medical imaging. The majority of tracers monitoring cell death are designed to probe: (1) disturbances in membrane asymmetry, (2) reductions in the membrane energetic barrier, (3) changes in MMP, and (4) activation of apoptotic caspases. Although these phenomena were initially considered hallmarks of apoptosis, similar processes occur in other forms of cell death. Thus, most imaging probes are not selective for one particular form of cell death. Increased uptake of such probes may be the net result of cells dying by various mechanisms.

### 3.1 Membrane asymmetry

#### 3.1.1 Exposure of PE

Several imaging probes have been developed to monitor the translocation of PE to the outer leaflet of the cell membrane during apoptosis. A few lantibiotics have been radiolabeled and tested for imaging of exposed PE; these include cinnamycin and duramycin.

##### Cinnamycin

Cinnamycin (Ro09‐0198) is a small peptide (2,046 kDa, 19 amino acids) from a family of lantibiotics isolated from *Streptoverticillium cinnamoneus*, which binds selectively to PE.^81^ A few in vitro assays have been performed with the fluorescein‐streptavidin (SA)‐labeled cinnamycin derivative fluorescein‐SA‐Ro, the iodine‐125‐labeled derivative (^125^I)‐SA‐Ro, or the AF546‐SA‐biotin‐labeled derivative,^63^, ^77^, ^78^, ^167^ for results see Table 3.

**Table 3:** Probes targeting altered membrane asymmetry (radiolabeled lantibiotics and annexin)

Probe/label	Target/K_d_	Preclinical evaluation	Human studies	Perspectives
Cinnamycin ^125^I	Exposed PE 10–200 nM^299^	Accumulates in apoptotic blebs in a PE‐specific manner^78^.	None	Little evaluated, perhaps because of toxicity.^300^
Duramycin ^99m^Tc	Exposed PE 4 to 11 nM^170^	Jurkat cells 171. COLO205 (human colon carcinoma cell line), MDA‐MB‐231 (human breast adenocarcinoma cell line), HT29 (human colon adenocarcinoma cell line) xenografts.^301^, ^302^, ^303^, ^304^	None. A tracer production kit has been developed.^305^	Detects exposed PE in apoptotic cells and the early response of tumors to chemo‐ and radiotherapy (uptake seven‐ to 30‐fold increased).
Duramycin ^18^F	Exposed PE 11 to 21 nM^172^	S180 (mouse fibrosarcoma cell line) tumors, A549 (human lung adenocarcinoma cell line) and SPCA‐1 (human lung adenocarcinoma cell line) xenografts.^306^	None	Only moderate (1.5‐fold) increases in tumors treated with chemotherapy.
Annexin A5 ^99m^Tc (HYNIC, tricarbonyl and various other labeling methods) ^111^In	Exposed PS 1 to 7 nM^307^, ^308^	PC12 (rat pheochromocytoma cell line), SHSY5Y (human neuroblastoma cell line) cells^308^. Rodent models of chemotherapy^182^, ^309^, ^310^, ^311^, ^312^, ^313^, ^314^, ^315^, ^316^, ^317^, ^318^, ^319^, ^320^, radiotherapy,^312^, ^314^, ^315^ and photodynamic therapy.^321^ Only limited preclinical data are available for [^111^In]Annexin A5.^322^, ^323^, ^324^, ^325^, ^326^	Pilot study in 15 cancer patients.^327^ Studies in 29,^328^ 17,^329^ 16¸^330^ and 38 patients^331^ indicated significant correlations of the early increase of tracer uptake after chemo‐ or radiotherapy with treatment response during follow‐up. Even a single baseline scan may be useful to predict tumor response to subsequent therapy.^332^, ^333^	Detects exposed PS and the early response of tumors to antitumor therapy (uptake up to sixfold increased). Detected primary tumors but did not visualize most affected lymph nodes in a human study.^334^ Uptake in non‐necrotic tumors is correlated to TUNEL (TdT‐mediated‐dUTP nick end labeling) staining^335^ and Fas ligand expression.^336^ Acceptable test‐retest reproducibility in head and neck cancer.^337^ However, Annexin‐SPECT has not become routine in the clinical setting for reasons discussed in Section 3.1.2. Tricarbonyl labeling results in better chemical stability of the probe than HYNIC labeling and precludes isomerization.^338^, ^339^, ^340^, ^341^, ^342^ Site‐specific labeling increases probe affinity^342^, ^343^, ^344^, ^345^, ^346^, ^347^, ^348^, ^349^, ^350^, ^351^ and improves pharmacokinetics (less kidney retention).
Annexin A5 ^123^I, ^131^I	Exposed PS 7 nM^352^	Less renal uptake than [^99m^Tc]HYNIC‐Annexin A5.^353^, ^354^, ^355^, ^356^, ^357^, ^358^, ^359^, ^360^, ^361^, ^362^	None	Poor metabolic stability (rapid dehalogenation).^353^, ^354^, ^355^, ^356^, ^357^, ^358^, ^359^, ^360^, ^361^, ^362^
Annexin A5 ^18^F	Exposed PS 2 to 10 nM^363^, ^364^, ^365^, ^366^	Jurkat, TC32 (primitive neuroectodermal tumor cell line) cells.^367^, ^368^ UM‐SCC‐22B, A549 (human lung adenocarcinoma cell line) xenografts,^363^, ^369^ VX2 (rabbit anaplastic squamous cell carcinoma) tumors.^369^	None	Site‐specific ^18^F labeling increases probe affinity.^369^, ^370^ Uptake in tumors then up to 40‐fold increased after treatment.
Annexin B1 ^99m^Tc	Exposed PS 50 nM^371^	Hepatic, thymus apoptosis models in mice.^371^, ^372^	None	Increased uptake correlates with histologic evidence of apoptosis. Probe injection may cause immune response.
Annexin B1 ^18^F	Exposed PS 10 nM^373^	Jurkat cells.^373^ W256 (Walker 256 carcinosarcoma cell line) tumors.^373^	None	Detects early response of tumors to chemotherapy (uptake sixfold increased). Risk of immune response.

HYNIC, hydrazinonicotinamide, Jurkat, immortalized line of human T lymphocytes.

##### Duramycin

Duramycin (PA48009, a peptide of 2,013 kDa and 19 amino acids) differs from cinnamycin by only one amino acid residue: Lys2 → Arg2.^168^, ^169^ Duramycin takes its name from being resistant to high temperatures and proteolysis. Soon after its discovery, duramycin was shown to interact with biological membranes and to have a high affinity (K_d_, 4–11 nM) to PE.^170^ The PE binding is specific and occurs in an equimolar and Ca^2+^‐independent manner.^171^ Duramycin binding to PE depends on membrane curvature and may alter both the curvature and permeability of the membrane. The mechanism by which duramycin induces these changes is unknown.^172^ Studies of protein domains involved in membrane tubulation and vesicle formation (e.g., ENTH [epsin NH2‐terminal homology] and BAR [protein dimerization domain named after the proteins Bin, Amphiphysin, and Rvs] domains) may provide clues on how duramycin can fold the membrane.^173^

The results presented in Table 3 suggest that radiolabeled duramycin but not cinnamycin is suitable for SPECT imaging of exposed PE. However, the tracer has not yet been tested in patients or in healthy human volunteers.

#### 3.1.2 Exposure of PS

Since PS exposure accompanies apoptosis, PS has been extensively studied as a target for the imaging of dying cells. Thus far, five families of protein or peptide‐based PS imaging probes have been employed: Annexin A5, the C_2A_ domain of synaptotagmin I, lactadherin, PS‐binding peptide 6, and bavituximab. Annexin A5 is the only probe that has proceeded to the clinical stage of testing. Imaging data for probes targeting exposed PS are presented in Tables 3 and 4.

**Table 4:** Probes targeting altered membrane asymmetry (other than lantibiotics and annexin)

Probe/label	Target/affinity	Preclinical evaluation	Human studies	Perspectives
C_2A_‐GST ^99m^Tc	Anionic phospholipids (PS) IC_50_ 90 nM^374^	Jurkat cells.^374^ H460 (human nonsmall‐cell lung cancer cell line) xenografts.^375^	None	Can be used to visualize and quantify apoptosis after chemotherapy.
C_2A_‐GST ^18^F	As above. IC_50_ unknown.	Jurkat cells.^202^ VX2 (rabbit anaplastic squamous cell carcinoma) tumors in rabbits.^202^ Uptake similar to [^18^F]Annexin A5.	None	As above. Strong increase after chemotherapy (>50‐fold). Probe can cross the blood–brain barrier.
C_2A_‐cH ^99m^Tc, ^111^In	As above. IC_50_ 55–71 nM^201^	Mouse models of lymphoma and human colorectal cancer.^204^	None. Kit‐based production possible.^376^	^99m^Tc‐labeled probe shows better tumor‐to‐muscle ratios than the ^111^In‐labeled derivative.
ATSE (diacetyl‐bis[N4‐ethylthiosemicarbazone])/AMal‐C_2A_c, ^64^Cu	As above. K_d_ 760 μM^377^	None (only radiochemistry reported).	None	^64^Cu offers longer physical half‐life than ^18^F. But labeling results in probe with very low affinity.
HYNIC (hydrazinonicotinamide)‐ lactadherin ^99m^Tc	Exposed PS. K_d_ sub‐nM^378^	HL60 cells,^212^ HeLa (human cervix carcinoma cell line) cells.^379^ Probe localizes mainly in the liver in mice and pigs.^378^, ^380^	None.	Binds in HL60 cells only to PS, but may in tissues also bind to integrins. Labeling of the C2 domain may result in a probe which is specific for PS.
PSBP‐6 ^99m^Tc	Exposed PS. K_d_ of Re analog 26 nM^381^	B16/F10 (mouse melanoma cell line) tumors.^381^ 38C13 (mouse B‐lymphoma cell line) xenografts.^382^	None	Can visualize apoptosis after chemotherapy.
NOTA (1,4,7‐triazacyclononane‐1,4,7‐triacetic acid)‐Ava‐PSBP‐6, ^64^Cu	Exposed PS. IC_50_ 23 μM^218^	EL4 cells.^218^ EL4‐tumors in mice.^218^	None	Very low affinity for PS because of labeling procedure, too low for successful imaging.
Bavituximab ^111^In	ß_2_‐glycoprotein 1 (binds to PS)	A549 (human lung adenocarcinoma cell line) xenografts.^383^ Impact of antitumor therapy not examined.	None	Labeled antibody visualized tumors and showed specific binding in SPECT.
Bavituximab ^74^As	ß_2_‐glycoprotein 1 (binds to PS)	Dunning R3227‐AT1 (Dunning prostate carcinoma) prostate tumors.^226^ Impact of antitumor therapy not examined.	None	Labeled antibody visualized tumors and showed specific binding in PET.
Bavituximab ^64^Cu	ß_2_‐glycoprotein 1 (binds to PS)	LNCaP (human prostate carcinoma cell line) xenografts.^384^	None	Labeled antibody visualized tumors in PET.
PGN635 ^89^Zr	ß_2_‐glycoprotein 1 (binds to PS)	KPL‐4 (human breast cancer cell line), COLO205 (human colon carcinoma cell line), HT29 (human colon adenocarcinoma cell line), and NCI‐H2122 (human nonsmall cell lung cancer cell line) xenografts.^385^	None	Seems useful for monitoring of the early response of tumors to chemo‐ or immunotherapy with PET.
PGN650 ^124^I	ß_2_‐glycoprotein 1 (binds to PS)	PC3 xenografts.^386^	Trial in 12 patients with advanced solid tumors (NCT 01632696). Results not yet reported.	In an animal model of prostate cancer, tumor‐to‐muscle ratios of radioactivity were inversely correlated with tumor growth measured during a follow‐up period of 28 days.^386^
KL15 betabody	Exposed PS	PC3 xenografts.^228^	None	Seems to bind also to (nonapoptotic) immune cells.

EL4, mouse lymphoma cell line; GST, glutathione S‐transferase; Jurkat, immortalized line of human T lymphocytes; PC3, human prostate carcinoma cell line.

##### Annexin A5

Annexin A5 (earlier called Annexin V or “placenta protein 4”) is an endogenous 36 kDa protein which was originally isolated from human placenta.^174^ Other tissues, such as endothelial cells, kidneys, myocardium, skeletal muscle, skin, red blood cells, platelets, and monocytes contain lower quantities of the protein.^175^ Annexin A5 was identified as a potent anticoagulant which could displace and inhibit coagulation factors from biological membranes.^176^ Its binding was attributed to a Ca^2+^‐dependent interaction with negatively‐charged PS molecules on the cell surface. Annexin A5 has no absolute specificity for PS, but binds with lower affinities to other targets, such as PE,^177^ membrane products of lipid peroxidation,^178^ vascular endothelial growth factor receptor 2,^179^ and integrin β5.^180^ For this reason, some Annexin A5 binding may be observed even in viable cells.

Although annexin A5 has been extensively tested in experimental animals and in cancer patients (see Table 3), for various reasons the original probe failed to meet clinical expectations^181^, ^182^, ^183^:

1. The radiolabeling procedures for Annexin A5 are rather elaborate and complex, which has limited application of the radiolabeled probe in a clinical setting.
2. Since Annexin A5 binds to exposed PS, an annexin scan cannot discriminate between apoptosis and necrosis. This caveat is true for all PS‐ and PE‐binding radiotracers. In a treatment response setting, the lack of specificity is not necessarily a problem, and may rather be an advantage, since PS‐ and PE‐probes can provide a stronger signal than pure apoptosis tracers and both apoptosis and necrosis can be desirable consequences of antitumor therapy.
3. Since the binding of Annexin A5 to exposed PS is calcium‐dependent, fluctuations (or regional differences) of intracellular Ca^2+^ concentrations may affect the binding of the tracer. This impact of calcium may result in high intraindividual variability of probe binding and an impaired test‐retest reproducibility of annexin scans.
4. The magnitude of Annexin A5 uptake in target lesions and the target‐to‐background (or signal‐to‐noise) ratios of Annexin A5 scans are usually rather low. Low uptake of the tracer may be partially due to poor penetration of Annexin A5 into tumor tissue. Poor image contrast may be caused by slow clearance of radiolabeled Annexin A5 from nontarget regions and blood, and by an increased uptake of the probe in normal tissues after antitumor therapy. In order to address this problem, Annexin V‐128 was developed, which shows a significantly lower kidney retention than Annexin A5 and is currently being evaluated in clinical trials.
5. High nonspecific accumulation of Annexin A5 in the liver and the kidneys makes it hard to detect tumors in the abdomen.
6. Tracer accumulation in areas far from known tumor sites may indicate the presence of unknown tumors or metastases, but may also be false positives, since Annexin A5 can accumulate in various benign lesions, such as infections and inflammations, capillary haemangioma, platelet‐rich thrombi, and unstable atherosclerotic plaques. Uptake of the tracer in such sites could be misinterpreted as indicating the presence of malignant lymph nodes.
7. The optimal timing of a post‐therapy Annexin A5 scan is frequently unknown or uncertain (which is true for all existing cell death‐targeting tracers), and a complex protocol with multiple scans may be necessary for correct evaluation of the response of a tumor to therapy. A protocol involving three separate injections of radiolabeled annexin and six whole‐body SPECT scans has been proposed for studies in cancer patients, in order not to miss an early response of the tumors to chemotherapy.^184^

##### Annexin B1

Annexin B1 is a PS‐binding protein isolated from the pork tapeworm (*Cysticercus cellulosae*, the larval stage of *Taenia solium*). The protein has a distinct N‐terminus and only 32 to 44% homology to other annexins, including Annexin A5.^185^ Radiolabeled Annexin B1 has been tested for SPECT and PET imaging of apoptosis (Table 3). [^99m^Tc]‐ and [^18^F]Annexin B1 showed predominantly renal clearance, like Annexin A5.

Although animal data indicate that apoptotic cells can be detected with radiolabeled Annexin B1, they have not demonstrated superiority of Annexin B1 over Annexin A5. Moreover, injection of a foreign protein like Annexin B1 may lead to an immune response in humans.

##### Zinc coordination complexes

Zinc‐dipicolylamine (Zn‐DPA) coordination complexes contain two *meta*‐oriented bivalent zinc cations and were created as mimetics to the domain of Annexin A5 which binds to PS via two bridging bivalent calcium cations.^186^ These small‐molecule complexes associate with negatively‐charged phosphorylated molecules, based on electrostatic interaction.^187^, ^188^ PSS‐380 has a binding site with high and a binding site with low affinity for Zn^2+^; coordination of the second Zn^2+^ molecule occurs only after association of the probe with the anionic membrane surface.^189^ PSS‐380 has only been used in an in vitro setting. In vitro and in vivo studies with a similar NIR probe (PSS‐794) demonstrated that Zn‐DPA complexes can detect human cells dying by apoptosis or necrosis, and bacterial infections.^190^, ^191^, ^192^, ^193^

The small molecular size of zinc coordination complexes could be an advantage and is one of the reasons why PET and SPECT analogues of these compounds were tested for apoptosis imaging (it could, e.g., lead to improved probe entry into tumor tissue). However, a high nonspecific binding of the labeled molecules in healthy tissue^194^ and/or a high uptake and retention of radioactivity in liver and intestines^195^, ^196^ was found to limit the usefulness of Zn‐DPA probes for visualization of cell death. Moreover, since Zn‐DPA complexes can bind to all kinds of anionic surfaces, positive SPECT or PET signals may not always reflect exposed PS.

##### Synaptotagmin I

Synaptotagmin I is a 65 kDa transmembrane protein primarily present in synaptic vesicles where it binds to negatively‐charged phospholipids in a Ca^2+^‐dependent manner to facilitate vesicle fusion and recycling during neurotransmitter release.^197^, ^198^, ^199^ The two cytoplasmic C_2_ domains (C_2_ **_A_** and C_2B_) of this protein have homology to PKC.^198^, ^199^ These domains interacting with Ca^2+^, phospholipids, and soluble *N*‐ethylmaleimide‐sensitive factor attachment protein receptor are involved in membrane fusion during synaptic vesicle cycling.^87^ Whereas the C_2A_ domain binds anionic phospholipids, such as PS (K_d_ = 15 – 40 nM) and phosphatidylinositol, the C_2B_ domain interacts with calmodulin and phosphatidylinositol.^200^ Imaging of apoptosis has been explored by labeling the 12 kDa C_2A_ domain with various fluorochromes, contrast agents (superparamagnetic iron oxide and Gd), and radionuclides (^99m^Tc and ^18^F). For this purpose, a C_2A_‐glutathione S‐transferase fusion protein was synthesized to prevent chemical modification in the PS‐binding site of C_2A_. Unfortunately, this approach yielded a heterogeneous probe mixture as any of the 14 Lys residues in C_2A_ could be labeled resulting in a decrease of affinity to PS. Therefore, a single‐site mutant of C_2A_ was developed (C_2A_m, S78C) with a Cys residue suitable for labeling and distant from the PS‐binding site.^201^

Initial experiments with a fluorescent probe showed that C_2A_ derivatives had much lower background binding in viable cells than Annexin A5 and were fourfold more specific in imaging cell death.^201^ However, since the affinity of C_2A_ for PS‐containing membranes (K_d_ = 20 to 71 nM) is much lower than that of Annexin A5 (K_d _=_ _1 to 7 nM), a >50 times higher protein concentration may be necessary for good images.^201^ The preclinical imaging results described in Table 4 have indicated that C_2A_‐based probes are potentially useful for evaluation of antitumor treatment, but have also some drawbacks:

1. High levels of radioactivity in liver, kidney, and abdomen may complicate the evaluation of tracer uptake in these areas, particularly at short intervals after injection. The C_2A_ domain labeled with ^18^F^202^ has shown a better clearance profile than the ^99m^Tc‐labeled analogue.^203^
2. Because of the large size of the C_2A_ molecule, tracer uptake is limited by the rate of diffusion into tissue. Radiochemists could try to produce probes with a reduced size and charge which may show a more rapid tissue entry.
3. Although in vitro experiments indicated a low background binding of C_2A_ derivatives in viable cells, target‐to‐background ratios of the radiolabeled compounds in the mammalian body were rather unfavorable. These low ratios could be related to a low affinity of the probes to PS‐containing membranes. C_2A_ domain probes with higher specificity and lower nonspecific retention have recently been developed, and as expected, these probes showed improved tumor‐to‐background ratios.^204^

##### Lactadherin (MFG‐E8, milk fat globule epidermal growth factor 8 protein)

MFGE8, a 46 kDa extracellular glycoprotein, is secreted by a subset of macrophages and dendritic cells. As a soluble molecule, it participates in the opsonization of apoptotic cells and their phagocytosis, adhesion between sperm and the egg coat, repair of intestinal mucosa, mammary gland branching, morphogenesis, and angiogenesis.^205^ The protein acts as a potent anticoagulant in blood^206^ and was linked to Alzheimer's disease and autoimmunity. It is a bridging molecule between apoptotic and phagocytic cells, has the ability to bind to integrins (α_v_β_3_ and α_v_β_5_) on immune cells via its arginylglycylaspartic acid motif of the glutamic acid‐leucine‐arginine domain,^207^ and also binds to membrane PS on apoptotic cells (with preference to PS in membrane areas of spiky morphology). The binding to membrane PS occurs via its F5/8‐type C1 and C2 domains (Kd_C1C2_ = 4.9 nM, Kd_C2_ = 2.0 nM) and does not require Ca^2+^. The C2 domain has about 100‐fold lower affinity toward soluble than membrane PS (Kd = 2.8 μM) and has a much higher affinity toward phosphatidyl‐L‐serine than phosphatidyl‐D‐serine.^208^ Despite functional similarity of the C‐domains present in synaptotagmin‐1 and lactadherin, they do not share any sequence homology,^207^ but there is homology between the C2 domains shared by lactadherin and blood coagulation factor VIII and V.^209^, ^210^ An in vitro study showed that lactadherin can specifically detect PS and has a higher affinity for PS than Annexin A5.^211^ Imaging data for a SPECT tracer based on bovine lactadherin are presented in Table 4.

Lactadherin binding to apoptotic HL60 cells was reported to be related to PS exposure and not to an interaction of the probe with integrins.^212^ However, the arginylglycylaspartic acid motif in lactadherin may bind to integrins throughout the body, which will likely complicate visualization of dying cells in living mammals. In future studies, lactadherin may be engineered in such a way that only the C2 domain, responsible for PS‐binding, is used for labeling. A fluorescent derivative of the C2 domain has shown the ability to label different cellular pools of PS^88^, ^92^ and apoptotic tumor cells.^208^, ^213^, ^214^

##### PS‐binding peptides

Using phage display technology, peptides were identified which can bind with considerable affinity to exposed PS. Clusters of the basic amino acids Arg (R) and Lys (K) appeared to be critical for (ionic?) interaction with this phospholipid. A peptide called PSBP‐6 has been radiolabeled for SPECT and PET imaging. The amino acid sequence of this peptide is based on the 14‐amino‐acid sequence from the C2 domain shared by PKC, PS decarboxylase, and synaptotagmin I.^215^

PS‐binding peptides are in theory an attractive alternative to PS‐binding proteins such as Annexin A5. The procedures for radiolabeling of peptides can be simpler, and the radioactive probes may show a more rapid entry into tumor tissue because of their smaller size. This reduced size can also result in a more rapid clearance of unbound probe from tissue and from blood. Moreover, peptides can be structurally modified, in order to improve their pharmacokinetic properties and metabolic stability. However, the currently available PS‐binding peptides seem to have insufficient affinity^216^, ^217^, ^218^, ^219^, ^220^ and/or specificity^221^ for their target phospholipid (see Table 4).

##### Bavituximab family of antibodies

An indirect option for imaging of externalized PS is provided by the generation of antibodies for β_2_‐glycoprotein 1. This protein is abundant in plasma and was shown to bind to negatively charged compounds, such as heparin, anionic phospholipids, and dextran sulfate. Two molecules of β_2_‐glycoprotein 1 are required for the interaction with PS (Kd ∼ 1 nM).^222^ Several murine monoclonal antibodies (e.g., 3G4 and 2aG4),^99^, ^223^, ^224^, ^225^ a chimeric monoclonal antibody (mAb) (bavituximab),^226^ and a human mAb (PGN635)^227^ were generated to detect PS exposure on tumor vessels. All of these antibodies have been explored preclinically and in clinical trials for treatment of different types of malignancy. Radiolabeled bavituximab, PGN635, and PGN650 have been used for noninvasive in vivo imaging of PS exposure (Table 4).

Bavituximab (MW = 145.3 kDa) was constructed by fusion of variable (Fν) regions from the mouse 3G4 antibody and human immunoglobulin G1κ constant regions. The chimeric antibody cross‐links and stabilizes a complex of two β_2_‐glycoprotein 1 molecules (Kd = 0.4 nM, MW ∼ 250 kDa) attached to the cell surface pool of PS.

PGN635 is a first‐in‐class PS‐targeting fully human mAb. The F(ab’)_2_ fragment of PGN635 was used to produce PGN650, which has similar affinity for PS‐β_2_‐glycoprotein 1 complexes as 3G4 and bavituximab.^227^

In an animal model of human prostate tumors, ^74^ As‐bavituximab displayed very high tumor‐to‐muscle ratios and specific binding in the tumor (Table 4). Nonvascular staining of dead and dying cells in and around necrotic tumor regions was observed only sporadically, which may indicate a poor ability of bavituximab to penetrate tumor tissue. If this is the case, antibody fragments, such as PGN650 may show better penetration. An open‐label, single‐arm clinical trial has been performed on 12 patients with advanced solid tumors, in which radioiodinated PGN650 was tested for tumor imaging, safety, and dosimetry. Unfortunately, the results of this trial have not yet been reported (Table 4).

##### ‘Betabodies’

’Betabodies’ are fusion products based on the PS‐binding domain(s) of β_2_‐glycoprotein 1 and the constant region of an antibody.^228^ The recombinant ‘betabody’ KL15 is expressed in a dimeric form and consists of the domain I and V from β_2_‐glycoprotein 1 fused with the CH2 and CH3 constant (Fν) domains of a mouse IgG2a antibody. Only a few preclinical data concerning this probe have been published (see Table 4).

### 3.2 Altered permeability of the cell membrane

#### 3.2.1 ApoSense family

**Figure 2:**
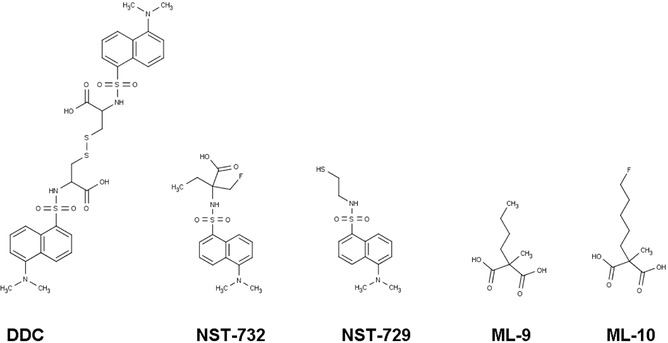
Chemical structures of members of the ApoSense family of compounds

The ApoSense family (Figure 2) is a group of small‐molecule compounds (size 300 to 700 D) that can be used to detect altered membrane permeability in apoptotic cells. The family comprises two different generations of molecules. *N,N*′‐Didansyl‐L‐cystine (DDC), (5‐dimethylamino)‐1‐napthtalene‐sulfonyl‐α‐ethyl‐fluoroalanine (NST‐732), and *N*‐(2‐mercaptoethyl)‐dansylamide (NST‐729) belong to the first generation. These molecules possess an amphiphatic structure, in which the hydrophobic moiety may provide a membrane anchor, while the charged moiety may prevent the compound from crossing healthy cell membranes. All contain a functional dansyl group with an inherent fluorescence. Butyl‐2‐methyl‐malonic acid (ML‐9) and pentyl‐2‐methyl‐malonic acid (ML‐10) belong to the second generation of the family. Their amphiphatic structure is based on an alkyl‐malonate motif, which is derived from γ‐carboxy‐glutamate‐rich Vitamin K‐dependent carboxylation/gamma‐carboxyglutamic protein domain‐containing proteins.^229^ Vitamin K‐dependent carboxylation/gamma‐carboxyglutamic protein domain containing proteins (e.g., growth arrest‐specific protein 6, coagulation factor X, vitamin K‐dependent protein S, and prothrombin) bind anionic phospholipids and calcium ions and are an important component of the blood clotting cascade.

ApoSense molecules were initially thought to detect both apoptotic and necrotic cell damage, but later studies have suggested that they specifically accumulate in apoptotic cells.^230^ Since ApoSense family members can cross the intact blood–brain barrier, they can be used to image the response of brain tumors to treatment, and loss of neurons after stroke or neurodegeneration in diseases like Alzheimer's disease. ApoSense compounds accumulate in the cytoplasm.^231^

Correlation between the in vitro uptake of DDC and Annexin A5 has suggested that scrambling processes in early apoptosis reduce the energetic barrier of the cell membrane and allow DDC to enter the cell. DDC uptake is thought to be the result of the following sequence of events:

Scrambling → membrane acidification → (mono)protonation of ApoSense molecules → flip‐flop of the molecule through the membrane by active scramblases and cell membrane depolarization → binding of the molecule to cytoplasmic proteins.

However, this proposed mechanism is not yet fully supported by experimental data. Imaging results acquired with ApoSense probes are summarized in Table 5.

**Table 5:** Probes targeting altered membrane permeability

Probe/label	Target	Preclinical evaluation	Human studies	Perspectives
DDC (fluorescent)	Membrane permeability (Ca^2+^ dependent, ATP‐independent uptake)	B16 (mouse melanoma cell line) tumors.^387^ Mouse model of chemotherapy‐induced enteropathy.^388^ Uptake is specific for cells involved in apoptosis.^388^	None (fluorescent probes are only suitable for studies in cells and experimental animals).	Detects response of tumors (and rapidly dividing cells) to chemotherapy (uptake up to sevenfold increased).
NST‐732 (fluorescent) ^18^F	Membrane permeability	Jurkat cells.^389^ LY‐S (mouse lymphoma cell line) tumors.^389^ Only results for fluorescent probe reported, not yet for the PET probe.^390^	None.	Detects response of tumors and tumor cells to radio‐ and immunotherapy (uptake up to 12‐fold increased).
NST‐729 (fluorescent)	Membrane permeability	Mouse models of Alzheimer's disease and ALS^391^	None (fluorescent probes are only suitable for studies in cells and experimental animals).	Co‐localizes with amyloid plaques in Alzheimer's disease and regions with axonal apoptosis in ALS.
ML‐9 ^3^H	Membrane permeability	Jurkat cells.^229^ CT26 (mouse undifferentiated colon carcinoma cell line) xenografts.^229^	None (ML‐9 cannot be labeled with a positron emitter. Its alkyl chain was modified in order to allow such labeling, resulting in the derivative ML‐10).	Detects response of tumors and tumor cells to chemo‐ and immunotherapy (uptake up to 10.6‐fold increased).
ML‐10 ^3^H ^18^F ^123^I	Membrane permeability	Jurkat cells: response to anti‐FAS mAb (monoclonal antibody) (ninefold increased uptake) is blocked by Z‐valine‐alanine‐DL‐aspartate‐fluoromethyl ketone and specific for cells involved in apoptosis.^392^ Colorectal tumor model with inbuilt doxocyclin‐induced “death switch”: strong apoptotic response clearly visible.^52^ [^123^I]ML‐10 is rapidly degraded in living animals.^393^	8 volunteers: [18F]ML‐10 is metabolically stable, radiation dose (3.6 mSv) is acceptable, but hydration and bladder catheterization are necessary during the scan.^392^ Ten patients with brain metastases, scanned shortly after radiotherapy: all lesions identified with MRI were detected, tracer uptake in tumors correlated with reduction of tumor size after 6–8 weeks.^394^	[^18^F]ML‐10 visualizes tumor response to therapy and may predict the therapeutic outcome. Accumulates in testes since spermatogenesis is accompanied by physiological apoptosis.

ALS, amyotrophic lateral sclerosis; Jurkat, immortalized line of human T lymphocytes.

Advantages of the Aposense family of compounds are: their small molecular size, the minimal number of functional groups, and the absence of chemically reactive, undesired labeling sites.^232^ Disadvantages are: the rather poorly defined mechanism of uptake and the requirement of a high administered dose. This last aspect raises concern about potential toxicity, since the dose is in the therapeutic rather than the tracer range. Some findings in animal models have suggested that the uptake of ML‐10 is pH‐sensitive.^233^ If ML‐10 uptake is indeed dependent on protonation, a decreased pH of the blood (e.g., due to failure of multiple organs after anti‐Fas antibody treatment) may result in a high nonspecific uptake of ML‐10 in viable tissues, whereas an increased extracellular pH (e.g., due to cyclophosphamide‐induced necrosis in treated tumors) could be associated with a decrease of ML‐10 uptake. Such factors may complicate the interpretation of PET images acquired with [^18^F]ML‐10.

### 3.3 Changes of mitochondrial transmembrane potential

Several lipophilic phosphonium cation‐based tracers (arylphosphonium salts) have been developed for in vivo imaging of treatment‐induced changes of MMP (Δψ_m_).^234^ Loss of negative charge at the inner mitochondrial membrane leads to reduced uptake of these lipophilic cationic tracers. Thus, radiolabeled arylphosphonium salts will generate a negative contrast.

#### 3.3.1 [^18^F]fluorobenzyl triphenyl phosphonium

[^18^F]fluorobenzyl triphenyl phosphonium (FBnTP) accumulates in cells with normal mitochondrial potential and washes out when this potential is impaired by apoptosis. When the baseline uptake of the tracer in tumor tissue is low, another imaging modality must be used for tumor localization.^235^ The signal of the tracer has been reported to be stable up to 45 min after injection.^236^ Changes in [^18^F]FBnTP uptake may be difficult to interpret since the accumulation of this tracer can be affected by cellular efflux processes driven by multidrug‐resistance proteins^237^ and by tissue‐dependent differences of background uptake. Various structural analogs of [^18^F]FBnTP have also been prepared, such as 4‐[^18^F]‐tetraphenylphosphonium (TPP),^238^, ^239^, ^240^ ([^18^F]fluoropentyl)triphenylphosphonium,^241^ and [^18^F]PEGylated‐BnTP.^242^ Uptake of these compounds is probably affected by the same processes as the tissue uptake of [^18^F]FBnTP.

#### 3.3.2 [^99m^Tc]sesta‐methoxyisobutylisonitrile

The SPECT perfusion tracer [^99m^Tc]sesta‐methoxyisobutylisonitrile (mibi) has been tested as a probe of reduced membrane potential in dying cells. An early study reported that the uptake of this tracer in human breast cancer cells (MCF7) was reduced when cells were treated with a cytostatic agent (sodium phenylacetate), and the decline of tracer uptake was correlated to the fraction of apoptotic cells.^243^ Another study reported that tumor uptake of [^99m^Tc]sestamibi was dose‐dependently reduced in mice bearing Ehrlich carcinomas that were subjected to radiotherapy. At 24 hr after irradiation, tumor‐to‐background ratios were inversely correlated with apoptosis index and the percentage of necrotic area, but at longer intervals (72 hr and 144 hr post irradiation) these ratios were inversely correlated only with the percentage of necrotic area.^244^ Although this study confirmed that [^99m^Tc]sestamibi is a “negative contrast tracer of dying cells,” another investigation performed in the same year showed that the absolute uptake values of [^99m^Tc]sestamibi in carcinomas are six‐ to eightfold smaller than those of a phosphonium cation like TPP.^238^ Thus, [^99m^Tc]sestamibi scans will show a considerably lower signal‐to‐noise ratio than TPP scans.

In summary: PET and SPECT probes of mitochondrial transmembrane potential have shown limited success. The uptake of such tracers is affected by the activity of transporters involved in multidrug resistance and by changes of the physical properties of target tissue. Changes in the uptake of such probes after antitumor therapy may not always reflect changes in mitochondrial transmembrane potential of tumor cells.

### 3.4 Increased proteolysis

Extrinsic and intrinsic apoptotic pathways converge at the level of caspase‐3 and caspase‐7 activation. The detection of activated caspases could be a valuable and specific tool for identifying dying cells before morphological features of cell death occur. Quantitative imaging of activated caspase‐3 and ‐7 may be more useful for monitoring tumor responses to therapy than for diagnosis and localization of unknown tumors. In vivo imaging of activated caspases is possible via two different approaches:

1. use of caspase inhibitors (Z‐valine‐alanine‐DL‐aspartate or isatin‐derivatives, for example, [^18^F]WC‐II‐89)^245^; and
2. use of caspase substrates (Z‐aspartate‐glutamate‐valine‐aspartate‐derivatives, for example, [^18^F]CP18).^246^, ^247^, ^248^

The main benefits of radiolabeled substrates over radiolabeled inhibitors are (in theory): (a) no problem of saturation of the binding sites, and: (b) signal amplification. Since a single enzyme molecule can convert several substrate molecules within the time frame of a PET or SPECT scan, the use of a substrate may result in a higher sensitivity for the detection of an active enzyme. However, in a comparative study between a caspase substrate and activity‐based probes (inhibitor‐based), signal amplification at the site of proteolysis did not have a dramatic enhancing effect on imaging. The authors believe that this was due to slow diffusion of the substrates into tissues and cells.^249^ In another study with inhibitor‐based probes, the abundance of active proteases in tumor tissues was found to be sufficient for the generation of images with acceptable contrast, therefore no saturation of binding sites occurred.^250^

#### 3.4.1 Caspase inhibitors

**Figure 3:**
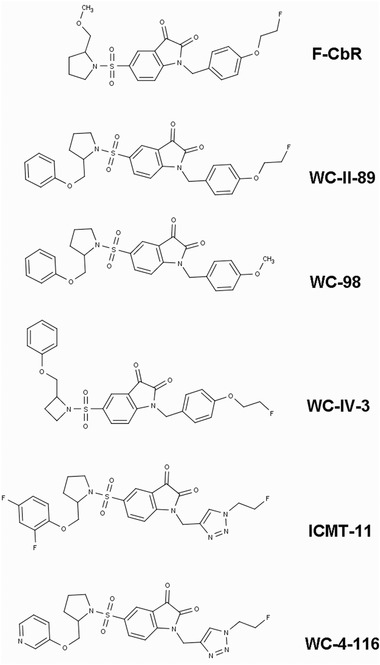
Chemical structures of radiolabeled isatins which have been tested as PET probes for caspase‐3

Radiolabeled inhibitors bind to a finite number of sites resulting in saturability of the probe binding.^251^, ^252^, ^253^ The amount of accumulation is dependent on the ratio of the concentration of active caspases and the affinity of the inhibitor for these caspases (B_max_/K_d_). The addition of a sulfonamide group confers isatins (i.e., derivatives of 1H‐indole‐2,3‐dione) a high affinity for caspase‐3 and ‐7.^254^ The chemical structures of some isatin‐based caspase inhibitors are shown in Figure 3, whereas imaging results acquired with these tracers are summarized in Table 6.

**Table 6:** Radiolabeled caspase inhibitors and substrates

Probe/label	Target/affinity	Preclinical evaluation	Human studies	Perspectives
IZ‐VAD‐fmk ^131^I	Pan‐caspase inhibitor Irreversible	Morris hepatoma cells.^395^	None	Low specific radioactivity, probe is mixture of two derivatives.
FB‐VAD‐fmk ^18^F	Pan‐caspase inhibitor, irreversible. IC_50_ 225 nM (caspase‐3).	SW620 (human colon carcinoma cell line), DLD‐1, COLO‐205, LIM‐2405 (human caecal adenocarcinoma cell line) xenografts.^396^	None	Detects activated caspases after chemotherapy (single, multidrug). Predicts later tumor shrinking.
CbR ^18^F	K_i_ 36 nM (caspase‐3), 93 nM (caspase‐7)	NMRI (Naval Medical Research Institute [mouse strain]) nude mice: rapid clearance from blood and plasma (within 10 min).^397^	None	Probably not useful. No further data reported.
WC‐II‐89 ^18^F	IC_50_ 9.7 nM (caspase‐3), 24 nM (caspase‐7)	Rodent models of hepatic apoptosis.^245^, ^398^ No tumor model data. Improved synthesis reported.^399^	None	Detects activated caspases in apoptotic cells.
WC‐98 ^11^C	IC_50_ 14.5 nM (caspase‐3), 22 nM (caspase‐7)	Rodent models of hepatic apoptosis. Observed increases were smaller than those of WC‐II‐89.^399^, ^400^	None	Probe detects activated caspases but WC‐II‐89 should be preferred.
WC‐IV‐3 ^18^F	IC_50_ 8.6 nM (caspase‐3), 26 nM (caspase‐7)	Rodent models of hepatic apoptosis.^245^, ^400^ Observed increases were smaller than those of WC‐II‐89.	None	Probe detects activated caspases but WC‐II‐89 should be preferred.
ICMT‐11 ^18^F	IC_50_ 0.5 nM (caspase‐3), 2.5 nM (caspase‐7)	RIF‐1, LNM35 (human pulmonary carcinoma cell line), PC9, A549 (human lung adenocarcinoma cell line) cells.^401^, ^402^ RIF‐1, 38C13 (mouse B‐lymphoma cell line), HCT116 (human colon carcinoma cell line), MDA‐MB‐231 (human breast adenocarcinoma cell line), PC9 tumors.^401^, ^402^, ^403^, ^404^ Probe detects activated caspases. For proper analysis of PET data in tumors with necrotic centers, voxel‐wise data analysis is a must.	Eight volunteers: eliminated via the hepatic and renal routes, acceptable dosimetry.^405^	Improved^406^ and GMP (good manufacturing practice) synthesis of [^18^F]ICMT‐11 have been developed.^407^, ^408^ Further evaluation of [^18^F]ICMT‐11 in patients receiving antitumor therapy is required.
WC‐4‐116 ^18^F	IC_50_ 4.5 nM (caspase‐3)	EL4 (mouse lymphoma cell line) cells.^409^ Colo205 (human colon carcinoma cell line) xenografts.^409^ Plasma levels and nonspecific binding of tracer increase after immunotherapy.	None	Pharmacokinetics seem problematic.
Tat_49‐57_‐γDEVDG‐NH_2_^131^I	Caspase‐3 substrate	Jurkat J6 cells.^255^ Peptide is fragmented; the radioactive G‐NH_2_ fragment is rapidly washed out.	None	Probe is not useful.
Tat_57‐49_‐γDEVDG‐NH_2_^131^I	Caspase‐3 substrate	Jurkat J6 cells.^255^ Peptide is not fragmented, but the product of caspase cleavage is rapidly washed out.	None	Probe is not useful.
CP18 ^18^F	Caspase‐3 substrate (highly selective)	U87MG (human glioblastoma cell line), A498 (human kidney carcinoma cell line), A427 (human pulmonary carcinoma cell line), LnCaP (human prostate carcinoma cell line), PC3 (human prostate carcinoma cell line), and Colo205 (human colon carcinoma cell line) xenografts.^410^, ^411^ Thymus apoptosis model.^412^ Mouse model of doxorubicin cardiotoxicity.^413^ CP18 may in colon cancer cells be a substrate for caspase‐3 and caspase‐9.^411^	Seven volunteers: cleared via kidneys, bladder dose can be reduced by frequent voiding^414^	Detects activated caspases after chemotherapy (see Figure 4). Considered suitable for patient studies.^414^

fmk, fluoromethyl ketone; Jurkat, immortalized line of human T lymphocytes; PC9, human lung adenocarcinoma cell line (differentiated); RIF‐1, murine radiation‐induced fibrosarcoma cell line.

Radiolabeled isatins have been shown to bind specifically to activated caspases, but their sensitivity as PET probes was limited. [^18^F]WC‐II‐89 may be better than [^11^C]WC‐98 or [^18^F]WC‐IV‐3 in discriminating the varying levels of active caspases in vivo. Although preclinical studies have indicated that [^18^F]ICMT‐11 has potential for evaluation of the impact of antitumor therapy, clinical application of this tracer is not very easy. Because of a low baseline uptake of radioactivity, tumor outlines cannot be assessed by PET but should be determined from a CT scan. The low baseline uptake may be considered as a favorable property of a cell death tracer, since in patients only small fractions of apoptotic cells are expected in tumor tissues at all posttreatment scanning intervals. Thus, the use of a CT or MRI scan will possibly be always necessary to delineate the tumors. Since radioactivity accumulates in liver, kidneys, intestines, and urinary bladder, assessment of the uptake of [^18^F]ICMT‐11 in abdominal tumors may be difficult or even impossible. Injected isatins can be trapped in blood (either due to apoptosis in lymphocytes, or to released, circulating caspases). Further optimization of the pharmacological properties of isatin‐based caspase inhibitors seems therefore necessary, but unfortunately, literature indicates that the list of chemical alternatives for existing caspase‐3/‐7 tracers is almost exhausted.

#### 3.4.2 Caspase substrates

The cellular trapping of radiolabeled caspase substrates is less sensitive to competition by physiological substances than the binding of radiolabeled caspase inhibitors, but intracellular retention of the cleaved substrate is necessary for successful imaging.

Currently used caspase substrates are based on the Z‐aspartate‐glutamate‐valine‐aspartate sequence. Since the inclusion of only a Aspartate, Glutamate, Valine, Aspartate, Glycine (DEVDG) or Asparagine, Glutamine, Valine, Asparagine, Glycine (NQVNG) amino acid sequence results in highly polar peptides, which do not cross cell membranes, some additional sequence should be attached to ensure membrane permeation. Membrane‐penetrating peptide sequences which could be explored are the following:

‐ Multiple Antigenic Peptide (MAP) peptide (X‐KLALKLALKALKAALKLA)—group 1, bilateral transport;
‐ transportan—group 1, bilateral transport;
‐ Tat—group 2, unilateral trapping, suitable for labeling because of the presence of Tyr;
‐ penetratin—group 2, unilateral trapping, not suitable for labeling because of the presence of Met.

In the first attempts at probe development, a Tat sequence (e.g., Tat49‐57, RKKRRQRRR) was added to ensure cellular uptake. It was demonstrated that insertion of yDEVDG at the C‐terminus of Tat was preferable, but the mechanism of uptake which is triggered by addition of that sequence is caspase‐independent.^255^

An elegant solution to the problem of intracellular retention of the cleaved substrate has recently been provided by the so‐called “smart probes” which display intramolecular macrocyclization and in situ nanoaggregation upon activation by caspase‐3.^256^, ^257^, ^258^ Due to sequence homology among the caspases, most caspase probes are not specific for caspase‐3 or caspase‐7. However, recent research on activity‐based probes has shown that the selectivity of such probes for a single caspase can be greatly improved by introducing several unnatural amino acids in the peptide recognition sequence.^259^, ^260^, ^261^, ^262^

**Figure 4:**
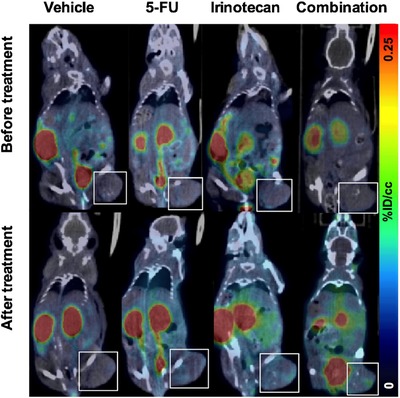
In vivo [^18^F]CP18 scans of tumor‐bearing mice (PET/CT images showing tracer uptake [%ID/cm^3^] in vehicle, 5‐FU (5‐fluorouracil), irinotecan, and combination‐treated animals (from left to right) before (upper panel) and after (lower panel) treatment. Tumors are indicated by white squares. Reproduced (with permission) from ref. 411

Imaging results acquired with radiolabeled caspase substrates are presented in Figure 4 and Table 6. Although the preclinical data presented in Table 6 (particularly those of [^18^F]CP18) have indicated that it is possible to image apoptosis and therapy‐induced increases of apoptosis with a radiolabeled substrate for caspase‐3, concentrations of radioactivity in target tissues were usually very low. Thus, the currently available caspase substrates seem to have not fulfilled their promise of significant signal amplification with respect to radiolabeled caspase inhibitors.

### 3.5 DNA damage and repair

As explained in Section 2.5 of this review, fragmentation of DNA is a process which accompanies both apoptosis and other forms of cell death. Environmental factors which may lead to the development of cancer, such as exposure to ultraviolet light, ionizing radiation, and carcinogenic substances, cause strand breaks in DNA. Moreover, many human cancers are characterized by deficiencies in DNA repair pathways compared to normal tissue. Finally, most forms of antitumor therapy induce damage to DNA. Many researchers have therefore attempted to develop radiopharmaceuticals which can visualize DNA damage and repair. Such tracers could be used to: (i) detect several forms of cancer at an early stage, (ii) evaluate the response of tumors to therapy, (iii) assess the biodistribution, pharmacokinetics, and target engagement of cytotoxic drugs aimed at inhibiting DNA repair, and (iv) select patients for treatment with such drugs. Results of attempts to visualize DNA damage and repair are summarized in Table 7.

**Table 7:** Probes targeting DNA damage and repair

Probe/label	Target	Preclinical evaluation	Human studies	Perspectives
PJ34 ^11^C	PARP‐1 (NAD^+^ binding site, activated enzyme)	Rat model of diabetes. Pancreatic uptake correlates with PARP‐1 expression in beta cells, reflects necrosis.^415^	None	Feasibility of PARP‐1 imaging demonstrated.
BO ^18^F	PARP‐1 (olaparib derivative, IC_50_ 17.9 nM)	MDA‐MB‐468, SKOV3, MIAPaCA‐2 (human pancreatic carcinoma cell line), PANC‐1 (human pancreatic epitheloid carcinoma cell line), A2780 (human ovarian carcinoma cell line) xenografts.^416^, ^417^, ^418^	None	Probe can quantify PARP‐1 expression in tumor cells and occupancy of PARP‐1 by olaparib.
PARPi‐fluorescein^18^F	PARP‐1 (olaparib derivative)	U87MG xenografts.^419^ Probe allows both PET and fluorescent imaging.	None	Rapidly defluorinated in vivo, thus not useful for in vivo studies.
PARPi ^18^F	PARP‐1 (olaparib derivative, IC_50_ 2.8 nM)	U251MG xenografts: >85% specific binding, tumor‐to‐brain ratios about 50. High uptake in lymph nodes and spleen because of PARP‐1 expression in immune cells.^420^	None	Good results. PARP‐1 tracers bind not only to tumor cells with DNA damage but also to inflammatory cells.
I2‐PARPi ^123^I, ^124^I, ^131^I	PARP‐1 (olaparib derivative, IC_50_ 9 nM)	U251MG, U87MG xenografts: 50–77% specific binding.^421^, ^422^	None	Probe visualizes target but shows smaller specific binding fraction than PARPi.
FTT (Fluor Thanatrace) ^18^F	PARP‐1 (IC_50_ 6.3 nM)	Genetically engineered fibroblasts.^423^ SNU‐251 (human ovarian carcinoma cell line), SKOV3 cells.^424^ MDA‐MB‐231 xenografts.^423^, ^425^ Probe does not bind to PARP‐2, shows specific binding in lymph nodes and spine.^426^	Radiation dose 6.9 mSv for 370 MBq, spleen and pancreas get highest dose.^426^	Probe is specific for PARP‐1. Can distinguish BRCA‐1 mutant from BRCA‐1 wild‐type cells after radiotherapy.
KX1 (FTT analog)^125^I	PARP‐1 (IC_50_ in nM range)	Genetically engineered fibroblasts, many cell lines.^427^ HCC1937 (human breast carcinoma cell line), MDA‐MB‐231 xenografts: considerable uptake but NO reduction after pretreatment of animals with cold olaparib.^427^	None	Probe is specific for PARP‐1. But its pharmacokinetics are not optimal (plasma levels of radioactivity and nonspecific binding are strongly increased after drug treatment; it is possibly a substrate for P‐gp [P‐glycoprotein]).
KX‐02‐019 (FTT analog)^125^I	PARP‐1 PARP‐2	Genetically engineered fibroblasts.^428^ EMT6 (mouse mammary carcinoma cell line) tumors.^428^	None	Probe is not specific for PARP‐1. Showed onkly moderate target‐to‐nontarget ratios.
Anti‐γH2AX‐TAT antibodies ^111^In	γH2AX	MDA‐MB‐468 cells.^429^ MDA‐MB‐468 xenografts.^429^ Transgenic mouse model of HER2 (human epidermal growth factor receptor 2)/neu overexpression‐driven breast cancer: tumor formation could be detected earlier with the SPECT probe (after 96 days) than with DCE‐MRI (dynamic contrast enhanced magnetic resonance imaging) (after 120 days) or by palpation (after 131 days).^430^	None	DNA damage response in tumor after radio‐ or chemotherapy can be visualized. SPECT with these antibodies has potential for early detection of malignant lesions.
Anti‐γH2AX‐TAT antibodies ^89^Zr	γH2AX	MDA‐MB‐468 cells.^431^ MDA‐MB‐468 xenografts: 2.4‐fold increased uptake after radiotherapy, about 60% specific binding.^431^	None	DNA damage response in tumor can be visualized.
ATRi ^18^F	ATR kinase	U251MG xenografts.^432^ Only minor decrease of target‐to‐nontarget ratio after target blocking.	None	Pharmacokinetics seem inappropriate for PET imaging.

BRCA1, breast cancer type 1 susceptibility protein; MDA‐MB, human breast adenocarcinoma cell line; SKOV3, human ovary adenocarcinoma cell line; U251MG, human glioblastoma cell line; U87MG, human glioblastoma cell line.

#### 3.5.1 Poly(ADP‐ribose) polymerase‐1

**Figure 5:**
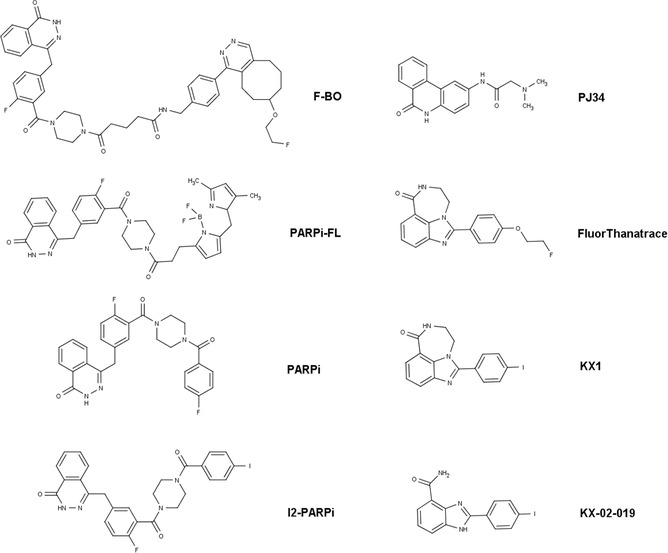
Chemical structures of radiolabeled inhibitors which have been proposed for imaging of activated PARP‐1

PARP‐1 is an enzyme in the nucleus of eukaryotic cells. When single‐strand breaks in DNA occur, PARP‐1 transfers ADP‐ribose units from NAD^+^ to various proteins, such as DNA polymerase and histones. This action of the enzyme plays an important initiating role in the repair of DNA, but when PARP‐1 is hyperactivated, cellular NAD^+^ pools are depleted, resulting in a decline of the levels of ATP and necrosis. Radiopharmaceuticals which target the expression or the activity of PARP‐1 have thus been used to evaluate target engagement of cytotoxic drugs. Such probes include radiolabeled analogs of the drug olaparib and derivatives of the benzimidazol carboxamide NU1085 (see Table 7 and Figure 5).

Some PET tracers for PARP‐1 have shown very promising results in animal models, particularly [^18^F]PARPi. However, all radiolabeled PARP inhibitors which have been studied thus far are hepatobiliary cleared. It remains to be seen whether the high accumulation of radioactivity in liver, intestines, and gall bladder constitutes a problem for application of these tracers in patients with abdominal cancer. The use of radiolabeled PARP‐1 inhibitors may be associated with two other complications: (i) Such probes may bind not only to dying tumor cells but also to immune cells, and (ii) DNA damage and repair will not always lead to cell death. Thus, PARP‐1 inhibitors will have a limited specificity for dying cells.

#### 3.5.2 Phosphorylated X isoform of the histone H2A (γH2AX)

When double‐strand breaks in DNA occur, the X‐form of histone H2A (H2AX) is phosphorylated (γH2AX) and several hundreds of phosphorylated protein molecules accumulate around each break site. The formation and accumulation of γH2AX is necessary for recruitment and activation of the subsequent processes of DNA repair. The expression levels of γH2AX are very low under normal physiological conditions, but show a strong and rapid rise after the induction of DNA damage. For this reason, γH2AX is an attractive target for SPECT and PET imaging. Imaging of this target may be used to visualize the impact of antitumor therapy.

Anti‐γH2AX antibodies can be used to quantify phosphorylated H2AX in permeabilized or lysed cells, but are not useful in living cells since such antibodies do not cross intact cell membranes. However, when the antibodies are linked to a cell penetrating peptide (“TAT sequence”), they are internalized in living cells and targeted to the nucleus (see Table 7).

A recent review on imaging of the DNA damage response^263^ concluded that several important issues still need to be addressed before anti‐γH2AX‐TAT antibodies can be applied in clinical studies:

1. A humanized version of the antibodies should be prepared, since the preclinically tested antibodies were raised in rabbits and will cause an immune response when they are injected in humans;
2. Since the currently used γH2AX‐TAT antibodies have a rather high nonspecific in vivo binding, it may be necessary to improve the target‐to‐nontarget ratio of these probes, for example, by using smaller antibody fragments rather than full antibodies, or by the application of a pretargeting strategy;
3. Quantification of the exact number of DNA double strand breaks may be difficult, since the local increase of γH2AX is not directly or linearly related to the number of strand breaks. More information about the biology of γH2AX is required to properly interpret PET or SPECT images acquired with anti‐γH2AX‐TAT.^263^

#### 3.5.3 Ataxia telangiectasia and Rad3‐related threonine serine kinase

Another important enzyme involved in the initiation and orchestration of the repair of DNA damage is ataxia telangiectasia and Rad3‐related threonine serine kinase (ATR kinase). A radiolabeled analog of the ATR kinase inhibitor Ve‐821 has been prepared but the results were disappointing (Table 7). Apparently, the pharmacokinetic properties of radiolabeled ATR kinase inhibitors need to be improved before they can be applied as PET tracers.

### 3.6 Other processes involved in cell death

**Figure 6:**
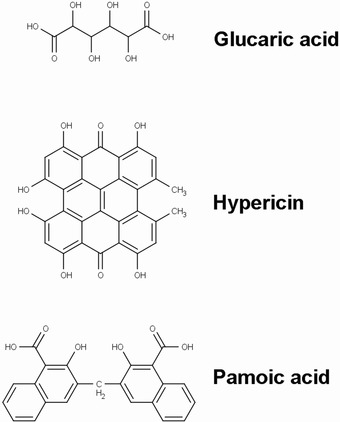
Chemical structures of some compounds which have been used to target tissue necrosis

Several imaging probes have been developed which may visualize necrosis. Imaging findings concerning these probes are summarized in Table 8 and the chemical structures of some probes are shown in Figure 6. The probes in question targeted the following processes:

**Table 8:** Probes targeting necrosis

Probe/label	Target	Preclinical evaluation/findings	Human studies/findings	Perspectives of the probe
ApoPep‐1 ^124^I, ^131^I	Histone H1	A549 (human lung adenocarcinoma cell line), H460 (human nonsmall‐cell lung cancer cell line) xenografts.^264^, ^433^ Can also be labeled with ^18^F for PET imaging.^434^	None	Potentially useful for imaging of cell death. Cyclic peptide may be better than linear one.^435^
3B9 mAb (monoclonal antibody) ^14^C, ^111^In	La autoantigen	EL4 (mouse lymphoma cell line) tumors.^436^	None	Detects tumor response to chemotherapy.^436^
Antimyosin ^111^In	Myosin heavy fragments	VX2, AH109A (rat hepatoma cell line) tumors^437^: Probe shows nonspecific accumulation in inflammatory cells in and around tumors.	Patients with rhabdomyosarcoma, leiomyosarcoma, and neurectodermal tumors.^438^, ^439^, ^440^, ^441^, ^442^ Tumors lacking the target (myosin) can also accumulate the probe!^443^	Probe is not a specific marker for necrosis, or release of myosin.
Glucarate ^99m^Tc	Unknown (histones?)	BT20, MCF7 (human breast cancer cell line), SUM190 (human breast cancer cell line), BxPC3 (human pancreatic adenocarcinoma cell line), HEK‐293 (human embryonic kidney cell line), and HCT‐116 xenografts.^444^, ^445^, ^446^, ^447^ Probe visualizes tumors and is not a substrate for P‐gp (P‐glycoprotein) or MRP‐1 (multidrug resistance‐associated protein 1).	Eleven patients with advanced head and neck cancer^448^ and 47 patients with lung or head and neck cancer: both primary lesions and metastatic sites visible.^449^ Tracer uptake after antitumor therapy correlated with later response.^449^	Probe may also accumulate in non‐necrotic ischemic, hypoxic, and/or hypoglycemic tissues.^450^, ^451^ Uptake mechanism is poorly defined.
Hypericin ^131^I ^64^Cu‐bis‐DOTA (1,4,7,10‐tetraazacyclododecane‐1,4,7,10‐tetraacetic acid)	Unknown but specific^452^ (may bind to cholesterol, PE, PS, and/or E‐DNA).^453^, ^454^	BT474 (human breast carcinoma cell line) xenografts,^455^ hepatic rhabdomyosarcomas,^456^ and VX2 tumors.^457^	Duodenal drainage catheter is required to reduce the intestinal radiation dose.^458^, ^459^, ^460^ The dose to thyroid and lungs is then still considerable.^461^ Intratumoral tracer administration has been proposed to circumvent these problems.^462^	Formation of aggregates should be avoided by addition of PEG400 or sodium cholate.^463^, ^464^, ^465^, ^466^ Various hypericin analogs with better solubility have been proposed.^454^, ^467^, ^468^, ^469^ Uptake mechanism is poorly defined.
Pamoic acid ^99m^Tc, ^68^Ga	Unknown	Animal models of hepatic infarction, hepatic necrosis, and muscle necrosis.^470^, ^471^, ^472^	None.	Uptake mechanism is poorly defined.

VX2, rabbit anaplastic squamous cell carcinoma.

#### 3.6.1 Exposure of histone H1

Apoptosis‐targeting peptide‐1 (ApoPep‐1), a hexapeptide identified by phage display, binds in a Ca^2+^‐independent manner to histone H1, which is exposed by apoptotic cells or becomes accessible in the nucleus of necrotic cells.^264^ The translocation of histone H1 during apoptosis proceeds in a caspase‐dependent manner and occurs at the early stage of apoptosis (before DNA fragmentation). The R3 residue was shown to determine binding and the ApoPep‐1 sequence was homologous to the G‐protein‐coupled receptor 83.

#### 3.6.2 Redistribution of La autoantigen

La autoantigen is a nuclear protein with an MW of 47 (or 48) kDa which is overexpressed in cancer cells with respect to cells of the tissue of origin. The La protein is cleaved by caspase‐3 during apoptosis, resulting in translocation of the NH_2_ terminus part of the molecule (MW 43 kDa) to the cytoplasm^265^ and accessibility of this part to anti‐La antibodies.^266^ Since the expression of the La autoantigen is selectively induced in dead or dying cancer cells after DNA‐damaging chemotherapy, imaging of this target is an interesting strategy for the detection of tumors and the evaluation of antitumor therapy.^267^

#### 3.6.3 Accessibility of myosin

Radiolabeled Fab fragments of monoclonal antibodies against myosin ([^111^In]antimyosin) have been widely used for the detection of myocardial cell injury and necrosis. Membrane disruption of myocytes makes it possible for such fragments to enter the dying cell and to interact with myosin heavy fragments.

#### 3.6.4 Exposed histones

[^99m^Tc]Glucarate ([^99m^Tc]‐D‐glucaric acid) is a six‐carbon dicarboxylic acid with a structural similarity to fructose. This SPECT tracer has been reported to accumulate in areas of acute ischemic injury where necrosis occurs, both within the brain^268^ and heart.^269^, ^270^, ^271^, ^272^ For this reason, [^99m^Tc]glucarate has also been tested as a cell death tracer in animal models of human cancer and in cancer patients (see Table 8).

#### 3.6.5 Extracellular DNA

Hypericin is a red pigment with antraquinone‐like structure (MW 504 Da), which has been isolated from St. John's wort *(Hypericum perforatum)*. Hypericin has been tested in many studies as a photosensitizer for photodynamic therapy. Since the compound accumulates in necrotic cells and tissues, hypericin has also been radioiodinated or labeled with ^64^Cu for the imaging of tumors and infarctions in experimental animals and humans (Table 8). Because of its polyphenolic polycyclic structure, hypericin has fluorescent properties and the compound can be detected in cell or preclinical experiments by optical imaging.^273^, ^274^, ^275^

#### 3.6.6 Unknown target (Pamoic acid derivatives)

The bis‐DTPA derivative of pamoic acid (4,4′‐methylenebis[3‐hydroxy‐2‐naphtoic acid]) is a necrosis avid contrast agent. The mechanism underlying accumulation of this compound in necrotic tissue is unknown.^276^ Various derivatives of pamoic acid have been radiolabeled and evaluated for visualization of necrosis with SPECT or PET (Table 8).

Unfortunately, most necrosis‐targeting probes seem to lack adequate specificity (see Table 8). They may accumulate in tissues by mechanisms unrelated to cell death (e.g., inflammation, ischemia, hypoxia, or hypoglycemia), and the uptake mechanism of these probes is poorly defined. Only the peptide ApoPep‐1 seems to deserve further evaluation.

## 4 CONCLUSIONS AND PERSPECTIVES

Although a large number of PET and SPECT probes for imaging of cell death have been developed, only a few radiopharmaceuticals have proceeded to the clinical stage of testing, viz. radiolabeled Annexin A5, PGN650, ML‐10, CP18, antimyosin antibodies, glucarate, and hypericin. Of these seven, the first four are the most likely candidates for translation to the clinic, and results of ongoing clinical trials with Annexin V‐124 and PGN650 are eagerly awaited.

An important issue concerning cell death imaging is the question whether radiopharmaceuticals should be specific for a particular death mode and biochemical process (e.g., activated caspase‐3 or caspase‐7), or can have limited specificity (e.g., detect exposed PS or anionic phospholipids). The required specificity will probably depend on the intended use of the tracer. In a basic science setting (visualizing of dying cells in animal and in vitro models of human disease), specificity of the used probe is very important in order to acquire specific information about the mechanisms underlying cell death (apoptotic vs. nonapoptotic, noninflammatory, or pro‐inflammatory, etc.). However, in a clinical setting (assessment of a patient's response to antitumor treatment), specificity of the probe may be of less importance. In this case, a probe with limited specificity that provides a stronger signal than a specific probe may be preferred. Here the main question to answer is whether cells have died. The question via which mechanism cell death was induced is then only a secondary issue.

In the extensive work performed with radiolabeled Annexin A5, two important difficulties were noted which will be of general concern in treatment response evaluation with any cell death tracer: (i) since the optimal timing of a post‐therapy scan is frequently unknown or uncertain, a complex (multi‐scan) protocol may be required for correct evaluation of tumor responses, and (ii) increases in cell death occur rapidly after the onset of therapy and correlate with early tumor shrinkage, but the magnitude of this early response to treatment is not always predictive for the long‐term response of a tumor. For a few tracers (i.e., [^99m^Tc]Annexin A5, [^18^F]ML‐10, [^18^F]FB‐VAD‐fluoromethyl ketone, [^99m^Tc]glucarate) and a few tumor models, data have been acquired demonstrating that the magnitude of early tracer uptake in the tumor corresponds to the extent of tumor shrinkage during follow‐up. There is definitely a need for more information about this subject, since valid predictive tools will allow clinicians to change therapy in nonresponding patients at an early stage, avoiding unnecessary toxicity and increasing treatment efficacy.

Since a limited probe entry into tumor tissue was frequently encountered in previous research (probably due to a large molecular size of the probes), radiolabeled protein domains or antibody fragments may be more promising as tracers than full‐length proteins or antibodies. Some novel potential tracer candidates have been identified in recent years, but have not yet been widely explored for PET and SPECT imaging. These include the Tim family of proteins which bind to PS via their IgV domain (but show a higher affinity to oxidized PS)^277^; Bai‐1, which binds to PS via thrombospondin domains^278^; and sRAGE, which binds PS via a V‐type domain.^279^ Other possible candidates are: antibodies against CXCL1, which is released during the unfolded protein response,^280^ the high mobility group box 1 (HMGB1) protein,which interacts with PS in an integrin‐dependent manner,^52^ and imaging of granzyme B, which may be a predictive biomarker of immunotherapy response.^281^ The already wide field of cell death imaging may thus expand even further in the near future.
